# Nanoencapsulation of Basil ( *Ocimum basilicum* L.) and *Echinophora platyloba* DC. Essential Oils in Chitosan Nanoparticles: Characterization and Efficacy in Burger Preservation

**DOI:** 10.1002/fsn3.72336

**Published:** 2026-09-06

**Authors:** Samira Malekiazar, Alireza Rahman, Mohammadreza Khani, Toktam Mostaghim, Abdolah Ghasemi Pirbalouti

**Affiliations:** ^1^ Department of Food Science and Technology, ShQ.C Islamic Azad University Shahr‐e Qods Iran; ^2^ Department of Food Science and Technology, TeMS.C Islamic Azad University Tehran Iran

**Keywords:** burger, chitosan, *Echinophora platyloba* DC, nanocapsules, sweet basil ( *Ocimum basilicum* L.)

## Abstract

Meat and its derivatives are susceptible to lipid and protein oxidation during processing and storage, negatively affecting product quality and stability. Oxidative processes can reduce nutritional value, flavor, texture, and shelf life. In this study, the effects of essential oils of two aromatic herbs [*Echinophora platyloba* DC. and basil ( *Ocimum basilicum* L.)] and their nanoencapsulation form (synthesized utilizing essential oils in conjunction with chitosan) on the physicochemical characteristics, antioxidant properties, microbial stability, and shelf life of burgers during storage at refrigeration temperatures were investigated. The particle sizes chitosan (without essential oil), chitosan nanocapsules with basil and *E. platyloba* essential oils were 500, 70.15, and 60.4 nm, respectively. The zeta potential and encapsulation efficiency of chitosan nanocapsules incorporating basil and *E. platyloba* essential oils were 63.61 and 65.18 mv, and 88.7% and 88.9%, respectively. The polydispersity index (PDI) values for chitosan nanocapsules, chitosan nanocapsules incorporating basil and *E. platyloba* essential oils were 0.711, 0.291, and 0.283, respectively. PDI of chitosan nanocapsules incorporating essential oils were lower than 0.5, indicating the uniform size distribution and thus the success of the nanoparticle production process. The chitosan nanocapsules with basil and *E. platyloba* essential oils could significantly enhance total phenolic content and antioxidant properties; however, pH, thiobarbituric acid (TBA) values, color changes, total microbial counts, particularly *Staphylococcus aureus* , as well as mold, yeast, and psychrotrophic bacterial populations meaningfully decreased. The chitosan nanocapsules with basil and *E. platyloba* essential oils (1:1) had an effect on reducing the microbial count by 76%, 78%, and 69% at 4, 8, and 12 days of storage period, respectively, compared to the control. The results of principal component analysis (PCA) and hierarchical cluster analysis (HCA) indicated that the nanoencapsulation of essential oils with chitosan could improve antioxidant stability, limit microbial growth, and maintain the physicochemical characteristics of burgers during storage. In conclusion, the incorporation of the *E. platyloba* and *O. basilicum* essential oils within nanocapsule structures offers a feasible approach for the development of novel, functional, and nutritionally advantageous products. This approach may also enhance shelf life, attributable to the inherent antimicrobial and antioxidant properties of the essential oils. Further studies are necessary for sensory evaluation of burgers, isolation of secondary metabolites, as well as toxic effects of nanoencapsulated bioactive compound formulations.

## Graphical abstract

Nanoencapsulation of the essential oils produced nanocapsules with small particle sizes and high encapsulation efficiency. Nanoencapsulation of the essential oils reduced microbial counts in the burger samples. The chitosan nanocapsules of essential oils enhanced total phenolic content and antioxidant properties in the burger samples. The nanoencapsulation of essential oils with chitosan could maintain the physicochemical characteristics of burgers during storage.

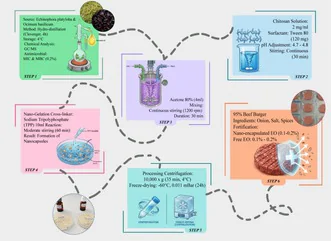

## 1 Introduction

Meat products contain diverse bioactive compounds vital for human physiological and biochemical balance. Burgers have gained popularity over other meat products due to their easy preparation, accessibility, nutritional benefits, cost‐effectiveness, and wide appeal. These products are essential to the human diet for their rich vitamins, proteins, and minerals (Alaei et al. 2023). Meat and its derivatives are susceptible to lipid and protein oxidation during processing and cold storage, negatively affecting product quality and stability. Oxidative processes can reduce nutritional value, flavor, texture, and shelf life (Bagher Abiri et al. 2026). Meats are prone to spoilage from pathogens, causing discoloration and producing volatile amines like ammonia and trimethylamine. These compounds indicate spoilage and are linked to pathogenic bacteria that can transmit disease through contaminated food (Zhang and Piao 2023). Nowadays, considerable investigation efforts are focused on decreasing or eliminating chemical additives in food by replacing them with natural substances. In this context, many research aims to identify natural antioxidants and antimicrobial agents derived from herbs to extend the shelf life and preserve the quality of meat and meat products (Khoshdouni Farahani et al. 2025).

Numerous bioactive secondary metabolites of plants are used in various industries like pharmaceutical, chemical, cosmetic, and particularly food industry. In recent years, there has been an increasing interest in the use of natural products due to concern for the safety of synthetic compounds, particularly antioxidant and antimicrobial agents. This challenge has led most research to focus on investigating biologically active compounds from plants with antimicrobial and antioxidant properties (Enteshari Najafabadi et al. 2024; Rezaei and Ghasemi Pirbalouti 2019). The essential oils, odorous, and volatile products belonging to plant secondary metabolites as important natural products have a wide range of applications in folk medicine, food flavoring, and preservation as well as in food industries (Farhadi et al. 2025). The essential oil constituents extracted from aromatic herbs have attracted considerable attention due to their diverse biological activities, particularly antimicrobial and antioxidant properties (Khwaza and Aderibigbe 2025). Additionally, the bioactive compounds of the herbs such as terpenoids, polyphenols, and flavonoids are valuable molecules that can reduce oxidative reactions, inhibit free radical formation, and suppress pathogenic bacteria in food (Farhadi et al. 2020; Rezaei and Ghasemi Pirbalouti 2019). In a sustainable food preservative system, adding antioxidant‐rich ingredients to meat products is highly beneficial. This practice extends product shelf life, preserves sensory attributes, and reduces lipid oxidation effects (Ebrahimi et al. 2022).

*Ocimum* L. (basil), belonging to the family Lamiaceae, comprises annual, perennial herbs and shrubs native to the tropical and subtropical regions. Sweet basil ( *O. basilicum* L.) is one of the key essential oil‐producing plants and one of the most widespread spices in the world, which is used as a culinary herb and widely cultivated in many countries (Ghasemi Pirbalouti et al. 2017). *O. basilicum* , as a remarkable aromatic plant, is grown in gardens, which the leaves and herbaceous parts of the plants are used as medicinal, vegetable, and culinary herbs. Both the fresh and dried leaves are widely used to enhance the flavor of foods like salads, meat, pasta, tomato products, pizza, soups, seafood, etc. (Ghasemi Pirbalouti, Mahdad, and Craker 2013). Iranian basil leaves are valued for essential oils in food applications; the seeds offer dietary fiber and potential as functional beverage ingredients (Farahani et al. 2026). Results of previous investigations (Ghasemi Pirbalouti, Mahdad, and Craker 2013; Gurkan and Hayaloglu 2023) indicated that methyl chavicol, methyl cinnamate, methyl eugenol, geraniol, citral, linalool, myrcene, limonene, thujone, caryophyllene, and farnesol are generally the major components of the essential oil of the leaves of basil. Due to its phenolic acids, flavonoids, terpenoids, and phenylpropanoids compounds, the essential oil and extracts of basil have antioxidant, antibacterial, antifungal, anti‐inflammatory, and antiviral properties, indicating that they may have significant advantages for human health (Ghasemi Pirbalouti, Mahdad, and Craker 2013).

The genus *Echinophora*, belonging to the family Apiaceae, has four species in the flora of Iran that are wild growing in central, northwestern, and western Iran (Ghasemi Pirbalouti and Gholipour 2016). *E. platyloba*, as an aromatic herb, is a perennial plant, which is well known for its favorable aromatic property (Moghaddam et al. 2015). The aerial parts of *E. platyloba* are used as flavoring agents in food products like pickled cauliflower, gherkin, yoghurt, cheese, etc. (Ghasemi Pirbalouti, Setayesh, et al. 2013). The presence of bioactive compounds such as terpenoids, polyphenols, and flavonoids in the essential oil and extract of this herb, due to antibacterial, antioxidant, and antifungal properties, can be critical in the context of food preservation and, finally, the promotion of human health (Ghasemi Pirbalouti, Setayesh, et al. 2013; Moghaddam et al. 2015).

Chitosan, a bioactive polysaccharide derived from chitin via deacetylation, has diverse applications. Chitosan has much utilization in sustainable food system that is used in food processing, packaging, and preservation. Chitosan gained significant interest for its safety, antifungal, antibacterial, biodegradability, biocompatibility, and antioxidant properties due to its rich amino and hydroxyl groups (Karimlar et al. 2026). The commercial value of chitosan is because of the valuable properties of its soluble derivatives, which are suitable in food processing, cosmetics, nano and biotechnology, and textile production (Ghasemi Pirbalouti et al. 2017; Rezaei‐Adl et al. 2025; Soltani et al. 2025).

Few food preservatives based on plant essential oils are already on the market. Additionally, an essential oil quantity greater than that required for the in vitro antimicrobial and antioxidant experiments must be added to the food to obtain an effective preservative activity (Granata et al. 2021). The essential oils are hydrophobic liquids that are volatile in nature and have a distinct odor. Despite the increased applicability of these bioactive compounds in diverse food, pharmaceutical, perfume, and health care industries, their lipophilic nature, unstable mixes, sensitivity to diverse environmental conditions such as oxygen, pressure, enzymatic activities, food interactions, pH changes, light, and temperature variations, as well as short effect over time, constrain their use in food and pharmaceutical applications. Hence, nanoencapsulation of the essential oils can be a valid and efficient strategy to surmount the above challenges (Suthan et al. 2025). The nanoencapsulation of the essential oils using chitosan can improve their bioaccessibility and bioavailability, protect them from degradation phenomena, boost solubility and physical stability, reduce volatility, and mask the intense aroma (Onyebuchi and Kavaz 2019). The nanocapsules have a subcellular size, a larger surface area per unit volume, and potential enhancement of essential oil concentration in food areas where the microorganisms are preferentially located. Furthermore, this technique helps maintain high levels of essential oil in food, enhancing their sensory qualities. It enhances the solubility and antioxidant properties of bioactive compounds and promotes controlled release (Tomé et al. 2023; Zhang and Piao 2023).

Generally, the nanocapsules are made up of emulsion layers, surfactants, and micelles that encapsulate the bioactive compounds, to reduce their loss on their way to the target site thereby maintaining the dosage and enhancing absorption, solubility, and reactivity. However, like every breakthrough, nanotechnology has its shortcomings, which are mainly associated with its unknown long‐term side toxicity effects on health and the environment in terms of residual toxicity and bioaccumulation (Verma and Pandey 2025). The shape, size, surface, composition, and charge of nanoparticles affect the safety of the formulation and thus determine the toxicity of the nanoencapsulated formulation. Results of previous studies illustrate that the functional groups present on the surface of nanoencapsulated formulation can determine the biocompatibility of the formulation (Kaur et al. 2024; Vieira and Conte‐Junior 2024). Therefore, it seems necessary to implement policies and regulatory considerations for the safe dosage, manufacturing method, and consumption rate of nanoencapsulated formulations (Food and Administration 2020).

The diverse uses of nanotechnology like nano‐capsulation in different pharmaceutical, food, and cosmetics products put forward a need for proper regulations to be implemented on nano‐products in use to ensure safety. Broad ranges of FDA‐approved nano‐products are in the market including medicines having high bioavailability, foods and food packaging. Regulatory considerations for applications of nanocapsules have been published by some countries. European Union and the United States Food and Drug Administration (US‐FDA) have published their regulatory principles (Verma and Pandey 2025). For instance, the European Union, the regulation of food labeling provides nano‐based food labels. Directives established emphasize that only safe products should be placed in markets (Jafari et al. 2017). The US‐FDA is the lead responsible agency for developing regulations for nano‐food products and follows the associated legislation in collaboration with the National Nanotechnology Initiative (NNI).

Meat consumption has soared in modern society due to its convenience in preparation. Meat is an ideal breeding ground for pathogenic bacteria and is prone to oxidative degradation, reducing its shelf life. Over time, it may develop odor, flavor, and color changes. To ensure consumer health, a variety of compounds must be used in beef burgers due to the ban on preservatives. To our knowledge, no documented studies have been reported to recognize the impacts of essential oils of *E. platyloba* and sweet basil and their nanoencapsulation form on the shelf life of beef burgers. Therefore, this study seeks to improve consumer health by adding essential oils from *E. platyloba* and sweet basil in free/nanoencapsulated forms with chitosan to beef burgers during refrigerated storage.

## 2 Materials and Methods

### 2.1 Chemicals and Reagents

Food‐grade chitosan used in this study was purchased from Sigma‐Aldrich (Steineheim, Germany). The DPPH (1,1–diphenyl–2–picryl–hydrazil), n‐hexane, TBA, and various culture media and reagents were purchased from Merck Co. (Darmstadt, Germany).

### 2.2 Plant Material

*Echinophora platyloba* DC. (Apiaceae) was collected from the alpine region of the Central Zagros Mountains range, Chaharmahal va Bakhtiari, Iran (2500 m above sea level, 32° N, 55° E), in summer 2023. Plant identify was confirmed by H.A. Shirmardi, Ph.D. (Research Center for Agriculture and Natural Resources, Iran). A voucher specimen of *E. platyloba* was preserved in the herbarium of the Research Center of Medicinal Plants, I.A.U., Shahrekord Branch, Iran (Herbarium specification No. IAUSHK‐157). A voucher specimen of sweet basil ( *Ocimum basilicum* L.) was located at the Herbarium of Research Institute of Forests and Rangelands of Iran (Code: RIFR 101598). After washing, the specimens were air‐dried at 25°C with 25%–28% moisture after that kept out of direct sunlight to maintain their aroma and fragrance.

### 2.3 Essential Oil Extraction

To extract the essential oil present in the aerial parts of *E. platyloba* (leaves, stems, and flowers) and basil (leaves and stems), the hydro‐distillation method was used. For this purpose, 100 g of the samples were transferred into a flask containing 1000 mL of water in a Clevenger apparatus (made by Glass Fabricating of Ashk‐e‐Shishe Co., Tehran, Iran) (Ghasemi Pirbalouti, Mahdad, and Craker 2013). The herbs and water mixture was boiled for two hours, after which the volume of the extracted essential oil was recorded. By opening the apparatus valve, the essential oil was transferred into dark‐colored bottles, and anhydrous sodium sulfate was added to the extracted essential oil.

### 2.4 Analysis of the Essential Oils

To identify the components of the essential oil, the essential oil obtained from each aromatic herb was injected into GC‐FID and GC/MS devices with the following specifications. GC was a Younglin Acme 6000 with a column length of 30 m, an internal diameter of 0.25 mm, and a film thickness of 0.25 μm of type BP5. To identify the components of the essential oil, the sample was diluted with *n*‐hexane and 1 μL was injected into GC. The temperature program for the column was set as follows: initial oven temperature of 50°C, held for 5 min; temperature gradient of 3°C/min; increased to 240°C, and then increased to 300°C at a rate of 15°C/min, with a hold at this temperature for 3 min, and a total response time of 75 min. GC/MS analyses of the samples were performed on an Agilent Technologies 6890 gas chromatograph coupled to an Agilent 5973 C mass selective detector and quadrupole EI mass analyzer (Agilent Technologies, Palo Alto, CA, USA) with a column length of 30 m, an internal diameter of 0.25 mm, and a film thickness of 0.25 μm of type BPX5. Mass spectra were recorded at 70 eV. Mass range was from *m/z* 40–500. For identifying the components of the essential oil, a sample diluted with *n*‐hexane was injected at a rate of 1 μL into the GC/MS device. The temperature program for the column was set the same as the GC method. Initial oven temperature was 50°C and programmed to increase at 3°C/min to 240°C and then increased to 300°C at a rate of 15°C/min. The injection chamber temperature was set at 290°C with a split ratio of 1–35. The FID detector temperature was set at 300°C, and helium gas was used as the carrier gas with a flow rate of 1 mL/min (Rezaei‐Adl et al. 2025). Helium gas was used as the carrier gas with a flow rate of 0.5 mL/min. The software used was Chemstation. The identification of the spectra was performed using their retention indices and compared with indices available in reference books and articles, using mass spectra of standard compounds and information available in the computer library (Adams and Sparkman 2007).

### 2.5 Nanoparticle Analysis

The particle size and polydispersity index (PDI) of the nanocapsules were measured using a Nano series Zetasizer, employing a helium‐neon laser at a controlled temperature of 25°C ± 0.1°C. For conducting measurements, the samples were prepared 1 mL ultrapurified water at neutral pH and subsequently positioned within a folded capillary cell. The data obtained from the device were analyzed utilizing the Malvern Zetasizer software. The properties of nanoparticles were measured at room temperature (Hashemi et al. 2023). The samples were subjected to three measurements for a single preparation.

### 2.6 Determination of the Antibacterial Activity

Two foodborne pathogens used in this study were *Staphylococcus aureus* (Gram‐positive, ATCC 25923) and *Escherichia coli* (Gram‐negative, ATCC 8739). The microorganisms were obtained from the Iranian Research Organization for Science and Technology. The bacteria were cultivated in Müller‐Hinton agar at 37°C for 18 h. The re‐culture was derived from the initial culture after that it was incubated at 37°C for 18 h. The second culture was mixed with sterile glycerin to create a 1:5 stock solution. The sample was stored in 100 μL aliquots at −20°C in tubes. The minimum inhibitory concentration (MIC) was determined using a micro‐dilution method with successive concentrations of essential oil (100, 125, 150, etc.) that were prepared in Müller‐Hinton medium with 5% dimethyl sulfoxide. In the method, 96‐well plates with circular apertures that have a 300 μL capacity. Next, 100 μL of essential oil was added to each well, followed by 20 μL of bacterial suspension. Each well was mixed for two minutes using a plate reader with a shaker. Light absorption was measured at 630 nm using a screen reader. The plates were incubated at 35°C for 24 h. After the incubation period, turbidity was assessed to determine the presence or absence of dimming in the wells. Light absorbance measurements were taken using a plate reader at different wavelengths. The results were compared with those of the commonly used antibiotic, ampicillin (Mahdavi et al. 2018).

Additionally, the determination of the antibacterial property of the essential oils was assessed using the disk diffusion assay. In this method, 10 μL of bacterial strains, activated 24 h prior to the experiment to a half McFarland standard, were inoculated onto Müller‐Hinton agar using an L‐shaped spreader. Paper disks (6 mm diameter) were impregnated with essential oil at various concentrations (5–40 mL/mg). The discs with different essential oil concentrations were aseptically placed on the culture medium using sterile forceps, ensuring firm contact by applying slight pressure. After a 24‐h incubation at 37°C, the inhibition zone diameter was measured in millimeters with a ruler (Enteshari Najafabadi et al. 2024).

### 2.7 Preparation of Nanocapsules Containing the Essential Oils

Nanocapsules were synthesized using ionic gelation. Total: 0.1 g of chitosan (deacetylation degree 85%–90%, molecular weight 200 kDa) was dissolved in 50 mL of 1% (*v/v*) acetic acid using a magnetic stirrer at 25°C. The resulting solution was centrifuged at 6000 rpm for 10 min to remove insoluble impurities, and the pH was adjusted to four using 1 N NaOH. This process prepared a chitosan solution at a concentration of 2 mg/mL. Subsequently, 120 mg of polysorbate (Tween 80) was added to the chitosan solution; after that, it was stirred continuously for 30 min for a homogeneous mixture. Each essential oil was mixed with 4 mL of 80% acetone at specific concentrations. The solution was added dropwise to the chitosan mixture while agitating at 1200 rpm for 30 min. Ten milliliters of a 4 mg/mL sodium tripolyphosphate solution at pH 4 was slowly added to the homogeneous emulsion while stirring moderately for 60 min under magnetic stirring at 250 rpm. The nanocapsules with the essential oils were centrifuged at 10,000 × g for 35 min at 4°C. The nanocapsules were rinsed with a 1% polysorbate (Tween 80) solution; after that, they were dispersed in distilled water. The final preparation was stored at 4°C until further use (Zhang et al. 2020).

### 2.8 Freeze‐Drying of Nanocapsules Containing the Essential Oils

Before freeze‐drying, the samples were frozen at −80°C for one hour. The primary drying occurred at −60°C for 24 h. The final drying phase was done at −75°C for one hour. The samples were kept in a low vacuum desiccator until use (Obradović et al. 2022).

### 2.9 Encapsulation Efficiency of Essential Oils Derived From the Herbs

To assess encapsulation efficiency, an initial volume of 1 mL from each sample was subjected to centrifugation at 13,000 rpm for a duration of 30 min to facilitate the separation of the capsules. Subsequently, to ensure the complete removal of particles that were not separated through centrifugation, the samples were subjected to filtration employing a 0.22 μm syringe filter. A volume of 40 μL from each sample was aliquoted and subsequently diluted to a final volume of 2 mL with methanol. Subsequently, the absorbance was measured using a UV–visible spectrophotometer at a wavelength of 275 nm.

The encapsulation efficiency was obtained using the equation:

$$\mathrm{EE}\;\left(\%\right)=\left[\left(\text{Total}\;\mathrm{EO}–\text{Free}\;\mathrm{EO}\right)/\text{Total}\;\mathrm{EO}\right]\times 100$$

where Total EO = Total essential oil used, Free EO = Free Essential Oil.

### 2.10 Morphological Characteristics of the Essential Oils Nanoencapsulated Using Chitosan

The surface morphology of nanocapsules was studied using a scanning electron microscope (MIRA3 FEG‐SEM, Tescan Co.; Czech Republic) at an accelerated voltage of 20 kV that was equipped with the TESCAN Essence Image Snapper software version 1.0.8.0. Scanning electron microscopy was employed to analyze the morphology and verify the dimensions at the nanoscale, specifically for features measuring less than 100 nm. A small aliquot of the sample was deposited onto an aluminum substrate utilizing silver adhesive. Subsequently, the substrate was subjected to a thin layer of gold deposition within a coating apparatus for a duration of 6 min to render the sample conductive. The specimens were subsequently transferred to a vacuum chamber for analysis. A beam of accelerated electrons was directed towards the samples, and an image was generated based on the electrons that were reflected from the samples (Rezaei Savadkouhi et al. 2020).

### 2.11 Preparation of the Burger Samples

The raw materials for the 95% burgers were procured and processed as per the formulation. Beef was sourced from a local market for this study. After separating the excess tallow, tendons, and bone fragments of the prepared meat pieces were weighed on a digital scale (model PX3000, Sanat Pand, Iran). Meats along with onions (5%) were ground using a meat grinder (Moulinex HV8, France). After that, 1% salt and 0.5% spice blend of black pepper, coriander seeds, and lemon powder were added to the onion and meat mixture. These ingredients were added using a precision digital scale (AND, GF‐600, Japan). A control sample (sample C) was provided using 100 g of burger dough composed of 95% without any essential oils. The concentrations of free and nano‐ nanoencapsulation of basil and *E. platyloba* essential oils were 100 and 200 mg per 1000 g of burger. The experimental treatments of free and encapsulated essential oils were added to the formulation, as per Table 1. The mixtures were molded to ensure uniform dimensions of 10 cm in diameter and 1 cm in thickness, which were then packaged and labeled in polyethylene bags. The packages were stored at 4°C for 12 days, with evaluations on Days 1, 4, 8, and 12 (Mozafari et al. 2023).

**TABLE 1:** The experimental treatments used in this study.

Experimental treatments	Free essential oil *E. platyloba* (%)	Free essential oil basil (%)	Nanoencapsulation of *E. platyloba* essential oil (%)	Nanoencapsulation of basil essential oil (%)
C	0	0	0	0
T1	0.2	0	0	0
T2	0	0	0.2	0
T3	0	0.2	0	0
T4	0	0	0	0.2
T5	0.1	0.1	0	0
T6	0	0	0.1	0.1

### 2.12 Physicochemical Analysis of Burger

The determination of moisture content in the raw burger samples was done utilizing the BM55S oven (Fan Azma Company), in accordance with the Iranian National Standard No. 745 (ISIRI 1971). Water activity was measured using an analyzer at a controlled temperature of 20°C on the unprocessed sample (Hartmann et al. 2020). Total phenolic content was measured using the Folin–Ciocalteu method, employing gallic acid as a standard, in accordance with the protocol established by Rezaei and Ghasemi Pirbalouti (2019). The absorbance was measured at 765 nm using an UV‐1280 spectrophotometer (Shimadzu, Japan). The absorption values were measured based on the standard curve. Gallic acid was used as the reference standard and the total phenolic content was expressed as mg of gallic acid equivalents per gram of each extract on dry basis (mg GAE/g extract) (Rezaei and Ghasemi Pirbalouti 2019). Total flavonoid content was approximated by aluminum chloride calorimetric assay based on the formation of a flavonoid–aluminum complex with a maximum absorptivity at 430 nm. The total flavonoid content is expressed as mg of quercetin equivalents per g of the sample. For determination of the antioxidant activity of the samples, different concentrations of the sample and ascorbic acid were prepared in three replicates, and DPPH solution was added to them (Rezaei and Ghasemi Pirbalouti 2019). In this assay, 3.9 mL of DPPH stokes, and 100 μL of the concentrations made for each sample was added and put in darkness for 30 min, and the absorbance was read at 517 nm at an UV‐1280 spectrophotometer (Shimadzu, Japan). DPPH radical inhibition using an equation:

$$\text{Scavenging effect}\;\left(\%\right)=\left[1-\left(A\;\text{sample}/A\;\text{control}\right)\right]\times 100$$

The pH of the burger samples was measured using the ZS‐200 pH meter manufactured by Zak Chemie, in accordance with the methodology established by Zhang et al. (2020). The lipid oxidation index was measured using the TBARS assay (Ebrahimi et al. 2022). The solution was homogenized for 1 min using a T‐25 homogenizer. The mixture was stirred for 10 min in a centrifuge at 5000 × *g* (Eppendorf 5424, Germany). The final product underwent cooling, after which absorption values at 532 nm were measured using a spectrophotometer (Shimadzu UV‐1280, Japan). TBARS values were determined using a standard curve (generated with 1,1,3,3‐tetraethoxypropane or MDA). Results are given as mg MDA/kg of sample (Ebrahimi et al. 2022). Color indices were assessed utilizing the HunterLab Colorflex (model D25‐DP900, Hunterlab, USA) (Alaei et al. 2023). Overall color changes (ΔE*) were computed using the subsequent formula.

$$\mathrm{\Delta E}=\sqrt{\Delta L^{2}+\Delta b^{2}+\Delta a^{2}}$$

### 2.13 Microbial Analysis of Burgers

The total microbial count was evaluated in accordance with the guidelines established by the Iranian National Standard No. 5272‐2. The culture medium employed in this study consisted of agar plates (ISIRI 2014). The enumeration of *Staphylococcus aureus* was conducted in accordance with the Iranian National Standard No. 6806‐3. The strain utilized in this experiment was cultivated on Parker agar medium (ISIRI 2006). Mold and yeast counts were performed in compliance with the Iranian National Standard No. 10899–1, the study utilized Dichloran Rose Bengal Chloramphenicol Agar (DRBC) medium for microbial analysis (ISIRI 2008). The enumeration of Salmonella was performed in accordance with the guidelines established by the National Standard (ISIRI 2006). The enumeration of *Escherichia coli* was carried out in compliance with the Iranian National Standard No. 2946 (ISIRI 2019). The quantification of psychotropic bacteria was conducted in accordance with the methodology established by Hasani‐Javanmardi et al. (2021). The implementation of agar plate culture medium was utilized (Hasani‐Javanmardi et al. 2021).

### 2.14 Statistical Analysis

The experiments were conducted using a completely randomized block design with seven treatments with three replications, including the essential oils in both free and nanocapsulated forms (using chitosan), applied individually and in combination (at equal ratios). Measurements were performed at four storage intervals (Days 1, 4, 8, and 12). All experiments were conducted in triplicate. Two‐way analysis of variance (two‐way ANOVA) and Duncan's multiple range test at a 95% confidence level (*p* ≤ 0.05) were performed using SPSS (ver. 24) software to determine the effects of treatments, storage periods, and their interactions on the physicochemical, antioxidant and microbiological properties of beef burgers. In the statistical models, treatments, storage periods, and their interactions were considered as fixed effects, while experimental replications were treated as random effects. Duncan's multiple range test was used for mean comparisons to assess the effects of treatments and storage. Data were expressed as mean ± standard deviation (SD), and statistical significance was accepted at *p* ≤ 0.05. Principal component analysis (PCA) and hierarchical cluster analysis (HCA) of sample variables during storage intervals were performed using R software (R: A Language and Environment for Statistical Computing).

## 3 Results and Discussion

### 3.1 Phytochemical of the Essential Oils

The essential oil from basil is a clear, colorless liquid. The chemical constituents of the essential oils of basil and *E. platyloba* were detected by GC‐FID and GC/MS. The profile of the essential oil extracted from sweet basil has shown in Table 2. Twenty‐two components were identified, accounting for about 97.5%. The major constituents identified were estragole or methyl chavicol (55.92%), 1,8‐cineole (10.33%), linalool (7.91%) and α‐cadinol<*Epi*‐ > (4.71%). The results demonstrated that the essential oil obtained from sweet basil contained monoterpene hydrocarbons (α‐pinene, β‐ocimene, etc.), phenylpropanoids (methyl chavicol), oxygenated monoterpenes (linalool, terpinen‐4‐ol, etc.), sesquiterpenes hydrocarbons (β‐caryophyllene), and oxygenated sesquiterpenes (α‐cadinol, β‐cubebene, etc.), confirming earlier reports that the main chemical groups obtained from the *O. basilicim* essential oil were phenylpropanoids and oxygenated monoterpenes (Ghasemi Pirbalouti, Mahdad, and Craker 2013; Ghasemi Pirbalouti et al. 2017; Moghaddam et al. 2015). An investigation into the antioxidant activity and phenolic content of the basil essential oil confirmed that methyl chavicol, linalool, and 1,8‐cineole are the main constituents (Ahmed et al. 2019).

**TABLE 2:** Chemical compositions of the sweet basil ( *Ocimum basilicum* L.) essential oil.

No.	Chemical compounds	RI_cal_ *	RI_ref_	(%)
1	α‐Pinene	935	939	1/34
2	*p*‐Cymene	1027	1026	0.63
3	1,8‐Cineole	1030	1031	10.33
4	*cis*‐β‐Ocimene	1047	1044	1/58
5	Fenchone	1088	1094	0/45
6	Linalool	1097	1098	7/91
7	Borneol	1163	1165	0.46
8	Terpinen‐4‐ol	1173	1177	0.89
9	Methyl chavicol or Estragole	1201	1195	55.92
10	β‐Elemene	1373	1375	0/58
11	Methyl eugenol	1399	1401	0.49
12	β‐Caryophyllene	1415	1428	1.2
13	Germacrene‐D	1474	1480	2
14	*cis*‐α‐Bisabolene	1518	1515	1.1
15	β‐Cubebene	1383	1390	2.42
16	γ‐Elemene	1447	1443	1.43
17	γ‐Cadinene	1526	1533	1/24
18	Elemol	1540	1547	0/51
19	Caryophyllene oxide	1573	1581	1/04
20	Cubenol	1621	1642	0/61
21	*α‐*Cadinol<*Epi*‐>	1643	1653	4/71
22	β‐Eudesmol	1651	1649	0/65

* RI, linear retention indices on DB‐5 MS column, experimentally determined using homolog series of C5–C24 *n*‐alkanes. RI lit, Relative retention indices taken from Adams.

In this study, 36 components were detected in the *E. platyloba* essential oil, accounting for about 91% (Table 3). The major constituents were *cis*‐β‐ocimene (22.66%), *p*‐cymene (5.47%), and α‐pinene (4.06%) belonging to the hydrocarbon monoterpenes; 1,8‐cineole (11.67%) and linalool (4.19%) are part of the oxygenated monoterpenes; methyl chavicol (10.12%) belonging to the phenylpropanoids (Table 3). In line with the results of this research, Akbari and Taherkhani (2024) reported that the major constitute of the essential oil extracted from *E. platyloba* (Shahrekord district, Iran) was β‐ocimene belonging to hydrocarbon monoterpenes. In another study by Mohammadi et al. (2025), the major compounds of the essential oils from the *E. platyloba* plants in natural ecosystems (Shahrekord district, Iran) under macronutrients treatment included β‐pinene, *cis*‐β‐ocimene, and *cis*‐sesquilavandulol.

**TABLE 3:** Chemical compositions of the essential oil of *E. platyloba*.

No.	Chemical compounds	RI_cal_ *	RI_ref_	(%)
1	β‐Thujene	1101	1102	0/51
2	α‐Pinene	935	939	4/06
3	β‐Pinene	977	980	1/34
4	Myrcene	993	991	3/89
5	4‐Carene	1001	1001	0/89
6	α‐Phellandrene	1003	1005	3/53
7	*p*‐Cymene	1026	1026	5/47
8	1,8‐Cineole	1029	1031	11/67
9	*cis*‐β‐Ocimene	1043	1044	22/66
10	γ‐Terpinen	1060	1062	0/47
11	α‐Terpinolene	1085	1088	0/45
12	α‐Pinene epoxide	1090	1095	0/24
13	Linalool	1096	1098	4/19
14	Borneol	1164	1163	0/55
15	Terpinen‐4‐ol	1173	1177	0/71
16	Isopinocampheol	1175	1178	0/51
17	*cis*‐Verbenol	1187	1188	0/82
18	α‐Terpineol	1192	1189	1/24
19	Myrtenol	1195	1194	0/6
20	Methyl chavicol or Estragole	1191	1195	10/12
21	Verbenone	1202	1204	0/35
22	Phellandral	1258	1251	0/66
23	Thymol	1285	1290	1.15
24	α‐Citral	1241	1240	1/81
25	Geraniol	1257	1255	1/21
26	Geranial	1269	1270	1.53
26	Isoledene	1370	1373	1.29
27	β‐Caryophyllene	1417	1428	1/49
28	α‐*cis*‐Bergamotene	1425	1430	0/35
29	γ‐Elemene	1446	1443	1.35
30	Elemol	1542	1547	0/71
31	(−)‐Spathulenol	1566	1575	2/04
32	Ledol	1561	1565	0/31
33	*cis*‐Sesquilavandulol	1632	1631	3.99
34	*tau*‐Cadinol	1637	1640	0/88
35	β‐Eudesmol	1652	1649	0/71
36	Geranylgeraniol	2023	2026	2/19

* RI, linear retention indices on DB‐5 MS column, experimentally determined using homolog series of C5–C24 *n*‐alkanes. RI lit, Relative retention indices taken from Adams.

In previous investigation (Sodeifian and Sajadian 2017), the major compounds of the essential oil extracted by supercritical carbon dioxide from *E. platyloba* (collected from Abadeh destrict, Iran) were linalool (16.02%), *trans*‐β‐Ocimene (11.58%), α‐pinene (7.10%), anisole, 2,4,6‐trimethyl (6.98%), and spathulenol (5.29%), while *cis*‐β‐ocimene (47%), spathulenol (9.0%), β‐myrcene (6.2%), limonene (5.4%), and α‐pinene (4.6%) were the main constituents of the *E. platyloba* essential oil by the hydro‐distillation method. In general, the chemical compositions of the essential oils from both herbs, varies significantly due to several factors. These factors include genetic factor, environmental conditions, and management factor (Bajalan et al. 2017, 2018; Farhadi et al. 2025; Ghasemi Pirbalouti et al. 2015; Momeni et al. 2023).

Although hydro‐distillation application could compromise the recovery of thermolabile compounds or those susceptible to hydrolysis, oxidation, or rearrangements, based on obtained results profile chemical compositions of the essential oil extracted by hydrodistlation had bit changes in the volatiles compounds.

### 3.2 Nanoparticles Characterization

Average particle size and PDI index are important and key factors that play a significant role in evaluating the efficiency of encapsulation, the release rate of the core active compounds and the stability of emulsions. Zeta potential has a strong relationship with the stability of particles and emulsions in colloidal systems (Mehrabi et al. 2026). According to our results, the average particle sizes of chitosan nanocapsules incorporating basil and *E. platyloba* essential oils were 70.15 and 60.4 nm, respectively (Table 4). In contrast, the average diameter for chitosan was 500 nm, indicating a larger particle size. This size reduction is likely to be due to the addition of the essential oils, which helps to evaluate their size and stability. The zeta potential of chitosan nanocapsules incorporating basil and *E. platyloba* essential oils were 63.61 ± 0.22 and 65.18 ± 0.18 mV, respectively (Table 4). Moreover, the PDI values for chitosan nanocapsules, chitosan nanocapsules incorporating basil and *E. platyloba* essential oils were 0.711, 0.291, and 0.283, respectively (Table 4), indicating a more uniform size distribution. The size index of nanocapsules is regarded as a significant determinant influencing the stability of microcarrier systems. The relative reduction in the size of nanocapsules enhances their effective surface area for the release of encapsulated compounds. Consequently, the dimensions of the nanocapsules generated in the current investigation appear to establish suitable textural properties in food products, exemplified by burgers. Similarity, results of a previous study (Đorđević et al. 2026) indicate that the nanoencapsulation of the basil essential oil was 177.4 nm, with increased stability and biological activity.

**TABLE 4:** Nanoparticle characterization parameters.

Samples	Z‐Average Diameter (nm)	Polydispersity Index (PDI)	Zeta potential (mV)	Encapsulation efficiency (%)
Nanoencapsulation of *E. platyloba* essential oil	60.4 ± 0.23	0.283	65.18 ± 0.18	88.948 ± 0.770*
Nanoencapsulation of basil essential oil	70.15 ± 0.39	0.291	63.61 ± 0.22	88.722 ± 0.207
Chitosan nanoparticles	500	0.711		—

* Mean values ± standard deviation.

Moreover, Đorđević et al. (2025) reported that the nanoencapsulation of a mixture of essential oils of basil and *Satureja montana* L. yields particles in the range of 150–200 nm. In another research (Onyebuchi and Kavaz 2019), a mean particle size of 134.9 nm was recorded for *Ocimum gratissimum* essential oil‐loaded chitosan nanoparticles. Chitosan‐based encapsulation of essential oils typically yields stable nanoparticles, indicated by a high positive zeta potential (+20 to +45 mV). The PDI value shows the homogeneity of droplet size distribution in emulsion systems, and it ranged from 0 to 1. Based on the standard classification, PDI values < 0.3 indicate a homogeneous particle size distribution, while values above 0.5 indicate a high degree of heterogeneity. Results of previous investigations indicated that the PDI values for chitosan nanoparticles without an active substance were in the range of 0.5 to 0.8, while the addition of essential oils usually decreased this value (Đorđević et al. 2025).

The findings regarding the encapsulation efficiency of essential oils derived from *E. platyloba* and basil plants are delineated in Table 4. There is no available literature data on the efficacy of encapsulation of the *E. platyloba* essential oil. High encapsulation efficiency protects the bioactive compounds like essential oils and enables their slow, controlled release (Đorđević et al. 2026).

### 3.3 Electron Microscopy Evaluation of Encapsulated Essential Oils

**FIGURE 1:**
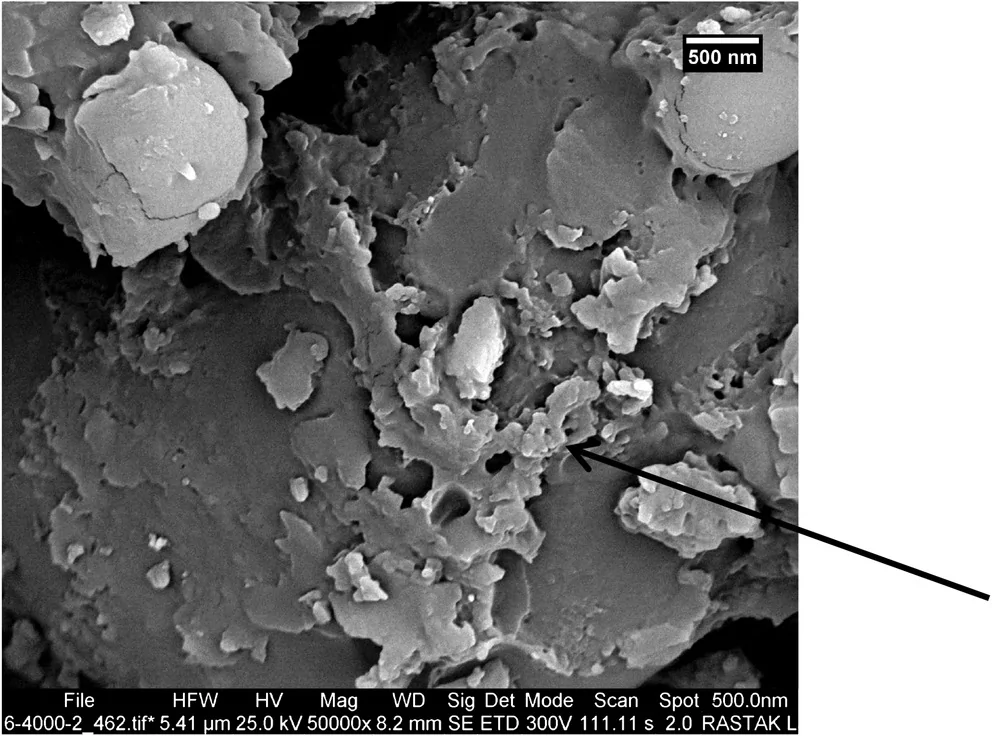
Electron microscope image illustrating the cross‐section of chitosan capsules encapsulating *E. platyloba* essential oil, at a magnification of 500 nm.

**FIGURE 2:**
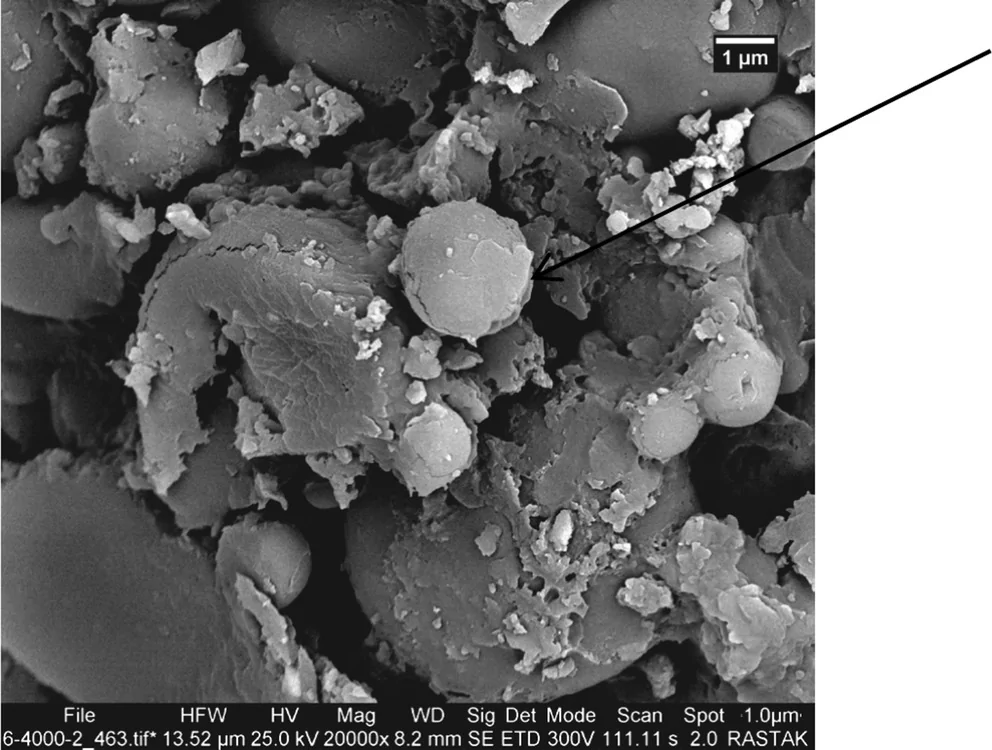
Electron microscope image depicting the cross‐sectional morphology of chitosan capsules that encapsulate *E. platyloba* essential oil, with a scale bar of 1 μm.

**FIGURE 3:**
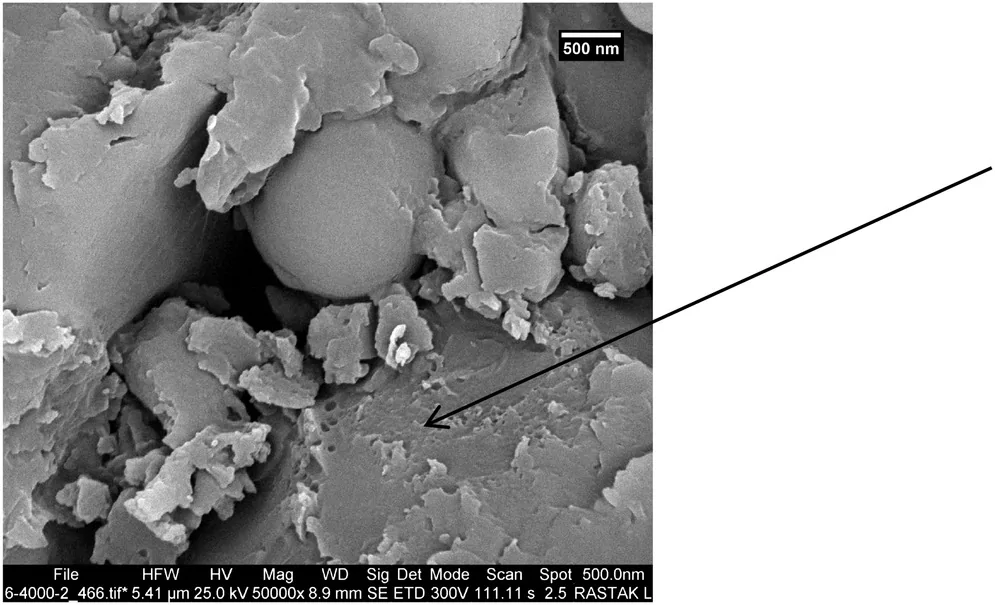
Electron microscope image illustrating the cross‐sectional view of chitosan capsules that encapsulate basil essential oil, with a scale bar of 500 nm.

**FIGURE 4:**
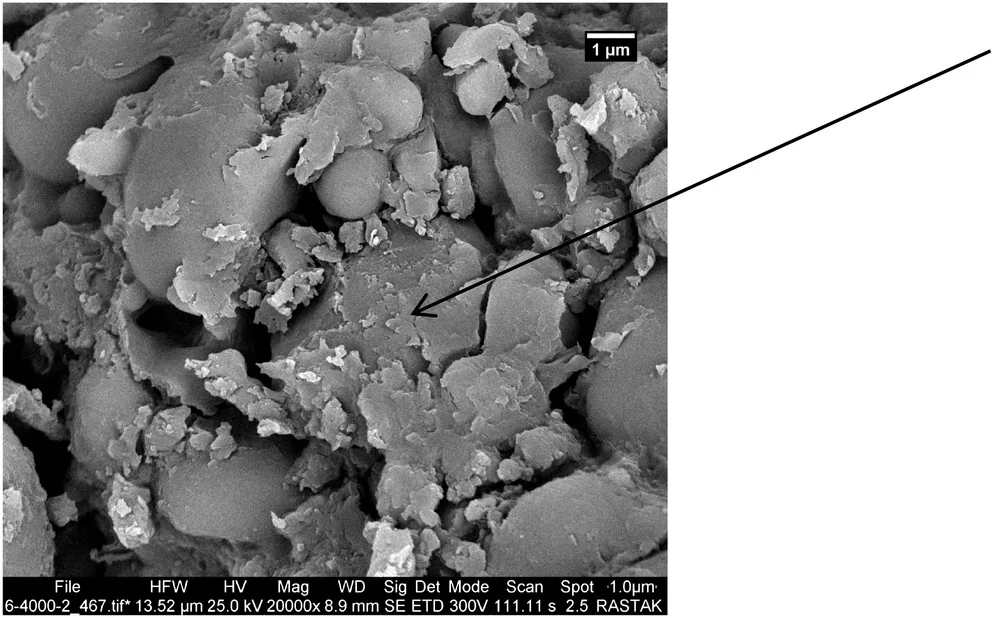
Electron microscope image depicting the cross‐sectional morphology of chitosan capsules that encapsulate basil essential oil, with a magnification of 1 μm.

Examining the microstructural characteristics of the nanocapsule matrix enhances our comprehension of its properties. In this study, the nanocapsules produced had an almost spherical shape with rough surfaces and surface depressions and had different sizes. The results indicated that this structure related to various microencapsulation techniques, such as spray or freeze‐drying that have an important impact on the physicochemical properties of obtained nano‐capsules from structural characterization and size. Hasani et al. (2018) reported that the nanocapsules based on chitosan and modified starch with lemon essential oil ( *Citrus limon* L.) as an active ingredient by freeze‐drying might cause structural changes in capsule morphology. Indeed, the capsules exhibit a smooth and compact microstructure, suggesting the establishment of a well‐organized matrix (Figures 1, 2, 3, 4).

Generally, the spherical shape of the capsules without cracks can protect the encapsulated oil from migration to the surface, exposure to oxygen and ultimately oxidation. The incorporation of essential oils was found to significantly enhance the roughness of the capsules while concurrently diminishing their cohesion. The essential oil in the capsule matrix has resulted in the development of a heterogeneous structure characterized by the presence of essential oil droplets dispersed within a continuous polysaccharide network. This phenomenon can be ascribed to the more pronounced structural characteristics of the capsules, as evidenced by the observed increase in film thickness. Additionally, the arrangement and spatial orientation of various molecules during the capsule formation process may result in diverse configurations, further contributing to these alterations in physical properties. The capsules exhibit a soft texture and possess a sponge‐like morphology, characterized by the presence of visible holes and pores throughout the entire cross‐section of the film, and the manifestation of holes and porosity within the cross‐sectional structure of the capsules is associated with the volatility characteristics of the essential oil. Indeed, these cavities represent regions that are occupied by essential oil that has volatilized from the surface of the capsules during the drying process.

### 3.4 Antibacterial Activity of the Essential Oils

Recent years have seen increased consumer awareness of health concerns, leading researchers to investigate natural antimicrobial compounds with minimal side effects. In the current study, the antimicrobial activity of the essential oils of *E. platyloba* and basil in two forms that is, free and encapsulation as natural antimicrobial compounds against *Staphylococcus aureus* and *Escherichia coli* was evaluated. Results of this research indicated that the highest diameter of inhibition zones against *S. aureus* and *E. coli* was obtained from free essential oil of *E. platyloba* in vitro condition (Table 5). According to results of the MIC and MBC values presented in Table 5, free essential oil of *E. platyloba* had the greatest antibacterial properties against *S. aureus* and *E. coli* . The essential oils are noted for their hydrophobic nature, a key characteristic linked to their antimicrobial properties. This property allows essential oils to interact with bacterial cell membranes and mitochondria, disrupting their structural integrity and permeability (Bajalan et al. 2017; Ghasemi Pirbalouti and Gholipour 2016; Khwaza and Aderibigbe 2025; Rezaei and Ghasemi Pirbalouti 2019). This disruption causes leakage of vital cellular components, ultimately leading to bacterial cell death. According to the results of MIC, MBC, and inhibition zone, *S. aureus* showed greater sensitivity to the natural antimicrobial compounds than *E*. *coli*. Gram‐positive microorganisms are more sensitive to antibacterial agents than Gram‐negative bacteria. The heightened sensitivity in Gram‐positive organisms is due to the lack of a lipopolysaccharide cell wall, which is present in Gram‐negative bacteria and helps block bioactive compounds from entering the cytoplasmic membrane.

**TABLE 5:** The antimicrobial properties (MIC, MBC, and diameter of inhibition zone) of the essential oils of *E. platyloba* and basil (mean ± standard deviation) against *S. aureus* and *E. coli* .

Experimental treatments	Diameter of inhibition zone *S. aureus* (mm)	Diameter of inhibition zone *E. coli* (mm)	MIC *S. aureus* (mg/mL)	MBC *S. aureus* (mg/mL)	MIC *E. coli* (mg/mL)	MBC *E. coli* (mg/mL)
Free essential oil of *E. platyloba*	13 ± 1	10 ± 1	0.390 ± 0.00	1.041 ± 0.45	1.041 ± 0.45	1.302 ± 0.45
Nanoencapsulation of *E. platyloba* essential oil	7 ± 1	6 ± 1	0.651 ± 0.23	1.302 ± 0.45	1.562 ± 0.00	1.562 ± 0.00
Free essential oil of basil	10 ± 1	9 ± 1	0.651 ± 0.23	1.562 ± 0.00	2.083 ± 0.90	1.562 ± 0.00
Nanoencapsulation of basil essential oil	6 ± 1	5 ± 1	0.781 ± 0.00	2.083 ± 0.90	2.604 ± 0.90	2.083 ± 0.90

Gram‐negative microorganisms resist antibacterial agents mainly due to their hydrophilic surfaces, which feature an outer membrane rich in lipopolysaccharides. This outer membrane acts as a barrier to antimicrobial substances. Periplasmic space proteins aid in degrading imported molecules, enhancing the antimicrobial resistance of these microorganisms. Gram‐positive bacteria lack an external membrane in their cell wall. Certain antimicrobial agents can disrupt the bacterial cell wall and membrane, causing the release of cytoplasmic contents (Rashidaie Abandansarie et al. 2019). Moreover, the essential oils can cross the bacterial membrane and alter the cytoplasm's pH and equilibrium of ionic concentration, leading to their antibacterial activity (Negi and Kesari 2022).

### 3.5 The Physicochemical Properties of the Burger Samples

Tables 6 and 7 show significant differences (*p* ≤ 0.05) in moisture content and water activity among the burger samples. The *E. platyloba* essential oil and chitosan‐nanoencapsulated of the basil essential oil had higher moisture content and water activity than other treatments Tables 6 and 7. All analyzed samples showed a decline in moisture content and water activity during the storage period. Humidity is a key factor in food quality. Chitosan nanocapsules with the essential oils effectively promote moisture retention. The essential oils improved the surface water repellency and bacteriostatic properties of the samples, leading to reduced water loss compared to the control group. This effect is likely due to the uniform distribution of smaller particles in the meat, which reduces evaporation of the encapsulated essential oil. The hydrophilic nature and high molecular weight of phenolic essential oil compounds help reduce layer softening and improve tensile strength, aiding moisture retention. Chitosan interacts with meat proteins, improving water retention and reducing water loss (Mahdavi et al. 2018). Chitosan nanocapsules with the essential oils significantly preserve moisture and improve product quality (Sun et al. 2021). The decrease in moisture content in the samples may be due to proteolytic enzymes converting protein substrates into free amino acids. This process reduces proteins' ability to retain moisture. Decline moisture content decreases soluble proteins, enhances oxidative changes, alters protein composition and color, resulting in lower product quality (Mahdavi et al. 2018). The preservation of aqueous activity may be attributed to the hydration capacity or water retention capability of chitosan employed in chitosan‐encapsulated nanotechnology treatments (Luciano et al. 2022). Moreover, the reduction in water activity is due to microbial and enzymatic processes that degrade proteins, lowering their solubility. The reduction in water activity of the burger samples with essential oils and nanocapsules may be due to phenolic compounds. These agents' antimicrobial properties reduce the microbial growth and protein degradation (Hemmatkhah et al. 2020).

**TABLE 6:** The moisture content of the burger samples containing free and encapsulated essential oils of *E. platyloba* and basil.

Storage time (Day)				
Experimental treatments	1	4	8	12
C	66.415 ± 0.088^A,ab^	64.166 ± 0.185^B,c^	62.788 ± 0.126^C,c^	61.890 ± 0.019^D,d^
T1	66.508 ± 0.390^A,a^	64.620 ± 0.316^B,b^	63.637 ± 0.198^C,b^	62.755 ± 0.114^D,b^
T2	66.346 ± 0.271^A,abc^	64.649 ± 0.204^B,b^	63.454 ± 0.076^C,b^	62.493 ± 0.231^D,c^
T3	65.792 ± 0.377^A,ab^	65.41 ± 0.096^B,a^	64.107 ± 0.145^C,a^	62.924 ± 0.055^D,b^
T4	66.150 ± 0.225^A,abc^	65.463 ± 0.365^B,a^	64.116 ± 0.262^C,a^	63.248 ± 0.093^D,a^
T5	66.373 ± 0.258^A,abc^	64.912 ± 0.170^B,b^	63.545 ± 0.347^C,b^	62.809 ± 0.236^D,b^
T6	65.750 ± 0.522^A,c^	65.551 ± 0.152^B,a^	64.264 ± 0.317^C,a^	63.231 ± 0.087^D,a^

*Note:* Significant different (capital) letters in each treatment indicate a significant difference between different days *p* < 0.05 according to Duncan test; Different lowercase letters in each day indicate that there is a significant difference between different treatments at *p* < 0.05 according to Duncan test; Values were expressed as means ± standard deviation (±SD).

**TABLE 7:** The water activity of the burger samples containing free and encapsulated essential oils of *E. platyloba* and basil.

Storage time (Day)				
Experimental treatments	1	4	8	12
C	0.936 ± 0.009^A,b^	0.925 ± 0.007^B,c^	0.911 ± 0.004^C,c^	0.902 ± 0.002^D,b^
T1	0.936 ± 0.004^A,b^	0.928 ± 0.006^B,bc^	0.921 ± 0.005^C,bc^	0.906 ± 0.003^D,b^
T2	0.939 ± 0.006^A,b^	0.929 ± 0.008^B,bc^	0.912 ± 0.007^C,c^	0.903 ± 0.008^D,b^
T3	0.957 ± 0.006^A,a^	0.942 ± 0.008^B,a^	0.933 ± 0.007^C,a^	0.919 ± 0.001^D,a^
T4	0.958 ± 0.009^A,a^	0.947 ± 0.004^B,a^	0.934 ± 0.012^C,a^	0.919 ± 0.003^D,a^
T5	0.942 ± 0.010^A,b^	0.925 ± 0.007^B,c^	0.912 ± 0.004^C,c^	0.906 ± 0.002^D,b^
T6	0.957 ± 0.011^A,a^	0.937 ± 0.005^B,ab^	0.931 ± 0.002^C,ab^	0.919 ± 0.004^D,a^

*Note:* Significant different (capital) letters in each treatment indicate a significant difference between different days *p* < 0.05 according to Duncan test; Different lowercase letters in each day indicate that there is a significant difference between different treatments at *p* < 0.05 according to Duncan test; Values were expressed as means ± standard deviation (±SD).

The duration of storage exerted a significant effect on the reduction of moisture content and water activity, with the control sample demonstrating a more pronounced decrease in both parameters. It is anticipated that the reduction in moisture content and water activity of the samples over the duration of storage can be attributed to the evaporation of water from the samples (Ghorbani et al. 2020). Moisture loss can result from the microbial and enzymatic activities that degrade proteins and reduce solubility. The reduced moisture in the burger samples with microcapsules of the essential oils may be due to the oils' migration and their antimicrobial properties. This effect likely inhibits microbial growth and prevents protein degradation (Hemmatkhah et al. 2020). However, results of an investigation (Mahdavi et al. 2018) have indicated that the moisture content of burgers decreased during storage when the samples were encapsulated in edible chitosan film enriched with the anise essential oil.

According to the results of this study, there were significant differences (*p* ≤ 0.05) in pH levels between the burger samples Table 8. There were no statistically significant differences found among the treatments on the first day of observation. At the end of the storage period, the highest pH was for the control, while the burgers containing 1% and 1% encapsulated essential oils of *E. platyloba* and basil had the lowest pH (Table 8). The increase in pH of the burgers during the storage period is related to biochemical changes caused by protein degradation and the production of volatile alkaline compounds such as nitrogen, amines, and hydrogen sulfide groups (Mehrabi et al. 2026). Moreover, the pH rise during storage is likely due to rapid degradation and the formation of alkaline compounds during bacterial growth, as well as the dissociation of carbonic acid. The samples treated with nanocapsules showed lower pH values than the control group. The reduction of pH may be due to the acidity of the acetic acid used to dissolve chitosan. The chitosan‐encapsulated nanocapsules showed a lower pH over storage, indicating potential enhanced antimicrobial activity. Chitosan can stabilize pH levels and inhibit microbial growth. The lower pH in samples with essential oils may be due to their antimicrobial properties (Zhang et al. 2020; Zhang and Piao 2023). The reduction of pH in the experimental treatments with the essential oils is due to their inhibitory effects on microbial growth. The antimicrobial properties of these essential oils reduce protein degradation, lowering alkaline compound production in burgers during storage (Ghorbani et al. 2024). Results of research show that the essential oils and chitosan coatings lower pH levels in the pork samples (Zhang et al. 2020). During storage under refrigeration and freezing, pH values significantly increased. The effect of storage duration on pH values was found to be statistically significant (Hemmatkhah et al. 2020).

**TABLE 8:** pH of the burger samples containing free and encapsulated essential oils of *E. platyloba* and basil.

Storage time (Day)				
Experimental treatments	1	4	8	12
C	5.79 ± 0.03^D,a^	6.19 ± 0.02^C,a^	6.56 ± 0.02^B,a^	6.99 ± 0.01^A,a^
T1	5.78 ± 0.04^D,a^	5.95 ± 0.02^C,c^	6.13 ± 0.02^B,d^	6.47 ± 0.02^A,d^
T2	5.79 ± 0.03^D,a^	6.11 ± 0.02^C,b^	6.35 ± 0.02^B,b^	6.74 ± 0.01^A,b^
T3	5.77 ± 0.05^D,ab^	5.85 ± 0.01^C,e^	5.93 ± 0.02^B,f^	6.15 ± 0.01^A,f^
T4	5.78 ± 0.04^D,a^	5.97 ± 0.03^C,c^	6.26 ± 0.02^B,c^	6.52 ± 0.02^A,c^
T5	5.78 ± 0.04^D,a^	5.90 ± 0.02^C,d^	6.08 ± 0.02^B,b^	6.35 ± 0.02^A,e^
T6	5.80 ± 0.02^D,a^	5.82 ± 0.01^C,f^	5.93 ± 0.03^B,f^	6.11 ± 0.01^A,g^

*Note:* Significant different (capital) letters in each treatment indicate a significant difference between different days *p* < 0.05 according to Duncan test; Different lowercase letters in each day indicate that there is a significant difference between different treatments at *p* < 0.05 according to Duncan test; Values were expressed as means ± standard deviation (±SD).

### 3.6 Antioxidant Activity of the Burger Samples

The antioxidant activity (based on DPPH scavenging activity) and total phenolic and total flavonoid contents are presented in Table 9. The findings reveal significant differences (*p* ≤ 0.05) among the burger samples. The *E. platyloba* essential oil enhances the antioxidant activity, evidenced by boosted DPPH scavenging activity and total phenolic and total flavonoid contents. Phenolic and flavonoids compounds are important natural antioxidant substances. The *E. platyloba* and basil essential oils possess antioxidant properties due to terpenoides particularly phenolic and phenylpropanoids compounds (Ghasemi Pirbalouti et al. 2017; Ghasemi Pirbalouti, Setayesh, et al. 2013; Moghaddam et al. 2015). Indeed, the antioxidant activity of the essential oils can be related by their impact on the structural attributes and levels of bioactive compounds, particularly terpenoides and phenolic acids (Ghasemi Pirbalouti, Setayesh, et al. 2013; Momeni et al. 2023; Oliveira et al. 2025; Rezaei and Ghasemi Pirbalouti 2019).

**TABLE 9:** DPPH (IC_50_; mg/mL) of the burger samples containing free and encapsulated essential oils of *E. platyloba* and basil.

Storage time (Day)				
Experimental treatments	1	4	8	12
C	42.92 ± 0.36^D,a^	49.31 ± 1.25^C,a^	57.52 ± 1.11^B,a^	74.98 ± 5.13^A,a^
T1	40.97 ± 0.56^D,ab^	43.98 ± 0.65^C,c^	46.73 ± 0.73^B,c^	54.41 ± 1.98^A,c^
T2	40.97 ± 0.56^D,b^	46.01 ± 0.71^C,b^	49.78 ± 1.66^B,b^	62.31 ± 1.30^A,b^
T3	32.99 ± 0.56^D,e^	36.03 ± 0.43^C,f^	39.18 ± 0.78^B,e^	41.53 ± 0.58^A,e^
T4	36.92 ± 0.46^D,c^	40.60 ± 0.85^C,d^	43.34 ± 0.63^B,d^	46.01 ± 0.71^A,d^
T5	42.31 ± 0.35^D,a^	44.41 ± 0.38^C,c^	45.55 ± 1.07^B,c^	49.31 ± 0.25^A,d^
T6	35.60 ± 0.42^D,d^	47.54 ± 0.72^C,e^	40.41 ± 0.55^B,e^	45.31 ± 0.69^A,de^

*Note:* Significant different (capital) letters in each treatment indicate a significant difference between different days *p* < 0.05 according to Duncan test; Different lowercase letters in each day indicate that there is a significant difference between different treatments at *p* < 0.05 according to Duncan test; Values were expressed as means ± standard deviation (±SD).

DPPH (IC_50_; mg/mL) of the burger samples containing free and encapsulated essential oils of *E. platyloba* and basil.

Initial analysis indicates that free essential oil samples contain high levels of primary phenolic compounds and flavonoids, which diminish over time during storage. Long storage reduced the antioxidant activity, as well as total phenolic and total flavonoid contents in all samples. Nano‐encapsulated treatments showed a smaller reduction in the antioxidant capacity compared to control and free essential oil treatments. The nanocapsules with the essential oil initially had lower phenolic compounds and flavonoids but enhanced over time (Tables 10 and 11). At the end of storage, the nanocapsule samples had higher total phenolic and flavonoid contents than the free essential oil in all treatments (Tables 10 and 11).

**TABLE 10:** The total phenol content (mg/g) of the burger samples containing free and encapsulated essential oils of *E. platyloba* and basil.

Storage time (Day)				
Experimental treatments	1	4	8	12
C	0.401 ± 0.004^A,d^	0.343 ± 0.002^B,g^	0.275 ± 0.004^C,f^	0.235 ± 0.001^D,f^
T1	0.484 ± 0.001^A,a^	0.465 ± 0.004^B,b^	0.445 ± 0.011^C,b^	0.419 ± 0.002^D,b^
T2	0.426 ± 0.005^A,c^	0.402 ± 0.004^B,f^	0.379 ± 0.008^C,f^	0.347 ± 0.002^D,e^
T3	0.489 ± 0.002^A,a^	0.470 ± 0.002^B,a^	0.458 ± 0.002^C,a^	0.429 ± 0.003^D,a^
T4	0.429 ± 0.003^A,e^	0.414 ± 0.001^B,e^	0.390 ± 0.003^C,e^	0.370 ± 0.001^D,d^
T5	0.469 ± 0.002^A,b^	0.441 ± 0.001^B,d^	0.413 ± 0.002^C,d^	0.379 ± 0.002^D,c^
T6	0.474 ± 0.006^A,b^	0.451 ± 0.002^B,c^	0.432 ± 0.002^C,c^	0.418 ± 0.002^D,b^

*Note:* Significant different (capital) letters in each treatment indicate a significant difference between different days *p* < 0.05 according to Duncan test; Different lowercase letters in each day indicate that there is a significant difference between different treatments at *p* < 0.05 according to Duncan test; Values were expressed as means ± standard deviation (±SD).

**TABLE 11:** The total flavonoids content (mg/g) of the burger samples containing free and encapsulated essential oils of *E. platyloba* and basil.

Storage time (Day)				
Experimental treatments	1	4	8	12
C	0.183 ± 0.001^A,g^	0.170 ± 0.001^B,f^	0.149 ± 0.001^C,f^	0.136 ± 0.000^D,f^
T1	0.201 ± 0.000^A,b^	0.196 ± 0.001^B,a^	0.189 ± 0.001^C,b^	0.177 ± 0.001^D,b^
T2	0.188 ± 0.001^A,f^	0.182 ± 0.001^B,e^	0.175 ± 0.001^C,e^	0.162 ± 0.000^D,e^
T3	0.203 ± 0.000^A,a^	0.196 ± 0.001^B,a^	0.190 ± 0.001^C,a^	0.181 ± 0.001^D,a^
T4	0.190 ± 0.000^A,e^	0.185 ± 0.001^B,d^	0.175 ± 0.001^C,e^	0.167 ± 0.000^D,d^
T5	0.197 ± 0.001^A,b^	0.190 ± 0.000^B,c^	0.181 ± 0.001^C,d^	0.169 ± 0.001^D,c^
T6	0.199 ± 0.001^A,c^	0.193 ± 0.001^B,b^	0.186 ± 0.001^C,c^	0.178 ± 0.001^D,b^

*Note:* Significant different (capital) letters in each treatment indicate a significant difference between different days *p* < 0.05 according to Duncan test; Different lowercase letters in each day indicate that there is a significant difference between different treatments at *p* < 0.05 according to Duncan test; Values were expressed as means ± standard deviation (±SD).

In this study, all treatments showed a significant decline in antioxidant activity as storage time increased. The initial increase in the antioxidant activity may be due to the essential oil's strong antioxidant properties. In later stages, this activity is likely influenced by factors such as increased oxidation, protein degradation, and pH fluctuations (Shukla et al. 2020). The increased antioxidant activity in the nano‐encapsulated samples during storage may result from the gradual release of the nanocapsules. This release mechanism extends shelf life, preserves nutritional quality, and reduces undesirable flavors in food. Plant bioactive compounds are released in control within the food matrix. The essential oils are released as the capsule wall degrades from oxidation and boost the membrane permeability. Storage conditions that increase lipid membrane fluidity enhance phenolic compound leakage. During storage, the water quantity around the microcapsules increases. This increase in water content enhances interactions between water molecules and the polar functional groups of the microcapsules. The nanoencapsulation of the essential oils can boost antioxidant capacity through enhancing some terpenoids like limonene, linalool, menthol, carvacrol, and thymol. These data illustrate that these biological compounds can also act as natural antioxidants, and the improved effect after nanoencapsulation may be related to the slow and gradual release of the active substances, greater solubility in food, and possible stability of temperature, light, and oxygen (Lelis et al. 2021).

The bioactive compound is released from the chitosan cavity into the surrounding space (Viacava et al. 2018). Indeed, chitosan can reduce free radicals due to its hydroxyl and carboxylic acid groups. Other researchers (Hemmatkhah et al. 2020) noted that the cumin essential oil boosts the antioxidant properties of the burger samples due to its phenolic compounds. Additionally, they reported that the cumin essential oils encapsulated in nanocapsules are released gradually over time (Hemmatkhah et al. 2020).

The experimental treatments had a significant impact (*p* ≤ 0.05) on the TBA content in the burger samples Table 12. The *E. platyloba* and basil essential oils had lower TBA levels than those with only basil essential oil. There were no significant differences found between the treatments on the first day. However, as storage time increased, the TBA levels rose in all samples. TBA quantification indicates the concentration of secondary oxidation products, affecting the product's sensory attributes. Consumers perceived pungency from oxidative processes at a threshold of 0.5 mg of malonaldehyde (MDA) per kg of meat products (Mozafari et al. 2023). Aldehyde compounds arise from the secondary oxidation of oils. These fatty acids oxidize extensively with oxidizing agents, forming unstable compounds like hydroperoxides. Unstable peroxides can be converted into more stable compounds, such as aldehydes. The aldehyde and ketone compounds result from advanced oxidation processes, measured by the TBA index (Rezvankhah et al. 2018). Increasing the TBA levels in the burger samples may result from oxygen exposure, leading to lipid oxidation. Reactive oxygen species from lipid oxidation accelerate the oxidation process (Xiong et al. 2020). The reduction in the TBA levels under the experimental treatments may be due to their strong antioxidant and regenerative properties.

**TABLE 12:** The thiobarbituric acid (TBA) (mg MD/kg) of the burger samples containing free and encapsulated essential oils of *E. platyloba* and basil.

Storage time (Day)				
Experimental treatments	1	4	8	12
C	0.169 ± 0.004^D,a^	0.241 ± 0.002^C,a^	0.310 ± 0.002^B,a^	0.360 ± 0.003^A,a^
T1	0.170 ± 0.001^D,a^	0.226 ± 0.002^C,d^	0.267 ± 0.007^B,c^	0.315 ± 0.003^A,c^
T2	0.168 ± 0.004^D,a^	0.234 ± 0.001^C,b^	0.285 ± 0.011^B,b^	0.330 ± 0.001^A,b^
T3	0.167 ± 0.004^D,ab^	0.222 ± 0.002^C,e^	0.247 ± 0.001^B,e^	0.281 ± 0.003^A,e^
T4	0.168 ± 0.007^D,a^	0.231 ± 0.002^C,c^	0.263 ± 0.002^B,cd^	0.316 ± 0.002^A,c^
T5	0.169 ± 0.007^D,a^	0.207 ± 0.002^C,f^	0.258 ± 0.001^B,d^	0.296 ± 0.002^A,d^
T6	0.168 ± 0.003^D,a^	0.201 ± 0.003^C,g^	0.237 ± 0.002^B,f^	0.268 ± 0.004^A,f^

*Note:* Significant different (capital) letters in each treatment indicate a significant difference between different days *p* < 0.05 according to Duncan test; Different lowercase letters in each day indicate that there is a significant difference between different treatments at *p* < 0.05 according to Duncan test; Values were expressed as means ± standard deviation (±SD).

Phenolic compounds in plants have strong antioxidant properties that inhibit MDA formation. This action delays lipid oxidation, potentially reducing lipid levels. The essential oil constituents show effective electron and proton donating properties. The adjacent radicals show notable stability due to electron delocalization in the benzene ring. This electron mobility makes the structure less vulnerable to oxidative attacks from molecular oxygen. The essential oils can neutralize free radicals and inhibit metal ions like ferrous ions (Fe^2+^). This inhibition reduces reactive oxygen species synthesis. Phenolic compounds in the essential oils may significantly inhibit oxidation during storage. The increase in the TBA levels during storage may be due to a lack of the antioxidant properties and a decrease in compounds concentration (Elsabagh et al. 2023; Mozafari et al. 2023). Extending storage to 12 days significantly increased oxidation levels in the burger samples. This increase was significantly reduced in the samples of the essential oils and nanocapsules compared to those with free essential oils. The results can be attributed to the degradation of free essential oils during storage, as well as the protective properties of nanocapsules in the burgers.

The findings indicate that controlled release of the flavonoids and phenolic compounds from the essential oil during storage is significant to these effects. Treatments with nanocapsules may gradually release antioxidant properties, extending their efficacy over time (Elsabagh et al. 2023, Mozafari et al. 2023). Consequently, it can be deduced that the process of encapsulation serves as a mechanism to inhibit the evaporation and degradation of the essential oils during storage. For instance, results of previous research showed that adding the rosemary essential oils and their nanoemulsion to beef burgers reduced the TBA levels. During storage, nanocapsules with the essential oils showed lower TBA levels than free essential oils and the control samples (Mozafari et al. 2023). Probably, there is a synergistic interaction or correlation between the phenolic content in the essential oil and TBA, which is responsible for the high antioxidant activity. In general, the results of some research that the nanoencapsulation of the essential oils offer a cost‐effective source of biological compounds that can be used as natural antioxidants in food and pharmaceutical formulae instead of synthetic antioxidants.

### 3.7 The Color Characteristics of the Burger Samples

The findings are in Tables 13, 14, 15, 16, detailing the color characteristics represented by L*, a*, b*, and ΔE. There were significant differences (*p* ≤ 0.05) in the color characteristics under the experimental treatments. The results indicate that longer storage reduces brightness and increases redness, jaundice, and ΔE in all samples. The *E. platyloba* and basil essential oils showed fewer color changes. Color is a key indicator of the visual appeal and quality in fresh meat. Consumers often use the redness of meat (a*) as a key indicator of freshness. Higher redness levels usually indicate fresher meat. Meat color is mainly influenced by myoglobin and hemoglobin pigments. Myoglobin is a key in determining meat color, closely linked to its redness (Hemmatkhah et al. 2020; Xiong et al. 2020).

**TABLE 13:** Brightness (L*) of the burger samples containing free and encapsulated essential oils of *E. platyloba* and basil.

Storage time (Day)				
Experimental treatments	1	4	8	12
C	53.32 ± 0.11^A,b^	52.27 ± 0.14^B,e^	51.31 ± 0.08^C,f^	50.28 ± 0.09^D,e^
T1	53.46 ± 0.13^A,b^	52.59 ± 0.16^B,d^	52.34 ± 0.38^C,d^	50.95 ± 0.37^D,d^
T2	53.38 ± 0.29^A,b^	52.55 ± 0.12^B,d^	51.74 ± 0.27^C,e^	50.97 ± 0.39^D,d^
T3	54.36 ± 0.11^A,a^	54.13 ± 0.08^B,b^	53.69 ± 0.16^C,b^	53.28 ± 0.10^D,b^
T4	54.34 ± 0.13^A,a^	54.03 ± 0.23^B,b^	53.26 ± 0.14^C,e^	53.00 ± 0.12^D,b^
T5	53.46 ± 0.13^A,b^	52.83 ± 0.05^B,c^	52.31 ± 0.12^C,d^	51.69 ± 0.61^D,c^
T6	54.27 ± 0.16^A,a^	54.47 ± 0.05^B,a^	54.17 ± 0.10^C,a^	53.84 ± 0.11^D,a^

*Note:* Significant different (capital) letters in each treatment indicate a significant difference between different days *p* < 0.05 according to Duncan test; Different lowercase letters in each day indicate that there is a significant difference between different treatments at *p* < 0.05 according to Duncan test; Values were expressed as means ± standard deviation (±SD).

**TABLE 14:** Redness (a*) of burger samples containing free and encapsulated essential oils of *E. platyloba* and basil.

Storage time (Day)				
Experimental treatments	1	4	8	12
C	13.59 ± 0.07^D,d^	14.35 ± 0.03^C,c^	14.92 ± 0.13^B,c^	15.33 ± 0.26^A,c^
T1	13.84 ± 0.08^D,c^	14.64 ± 0.21^C,c^	14.97 ± 0.24^B,c^	15.26 ± 0.07^A,c^
T2	13.74 ± 0.04^D,b^	14.50 ± 0.24^C,c^	14.94 ± 0.21^B,c^	15.27 ± 0.25^A,c^
T3	15.26 ± 0.13^D,a^	15.64 ± 0.14^C,a^	16.15 ± 0.14^B,a^	16.34 ± 0.08^A,b^
T4	15.32 ± 0.13^D,a^	15.82 ± 0.16^C,a^	16.13 ± 0.10^B,a^	16.67 ± 0.06^A,a^
T5	14.38 ± 0.03^D,b^	15.04 ± 0.03^C,b^	15.38 ± 0.06^B,b^	15.49 ± 0.21^A,e^
T6	15.33 ± 0.15^D,a^	15.60 ± 0.26^C,a^	16.03 ± 0.15^B,a^	16.28 ± 0.05^A,b^

*Note:* Significant different (capital) letters in each treatment indicate a significant difference between different days *p* < 0.05 according to Duncan test; Different lowercase letters in each day indicate that there is a significant difference between different treatments at *p* < 0.05 according to Duncan test; Values were expressed as means ± standard deviation (±SD).

**TABLE 15:** Jaundice (b*) of the burger samples containing free and encapsulated essential oils.

Storage time (Day)				
Experimental treatments	1	4	8	12
C	18.25 ± 0.11^D,b^	18.81 ± 0.16^C,c^	19.81 ± 0.05^B,b^	20.61 ± 0.12^A,a^
T1	18.34 ± 0.05^D,b^	18.54 ± 0.11^C,d^	19.19 ± 0.17^B,cd^	19.74 ± 0.13^A,c^
T2	18.31 ± 0.25^D,b^	18.50 ± 0.07^C,d^	19.36 ± 0.13^B,c^	20.17 ± 0.12^A,b^
T3	19.35 ± 0.10^D,a^	19.63 ± 0.09^C,a^	19.90 ± 0.13^B,c^	20.24 ± 0.09^A,b^
T4	19.44 ± 0.13^D,a^	19.75 ± 0.09^C,a^	20.09 ± 0.03^B,b^	20.52 ± 0.18^A,a^
T5	18.25 ± 0.04^D,b^	18.47 ± 0.06^C,d^	19.16 ± 0.06^B,d^	19.54 ± 0.08^A,d^
T6	19.23 ± 0.04^D,a^	19.46 ± 0.06^C,b^	19.78 ± 0.08^B,b^	20.08 ± 0.04^A,b^

*Note:* Significant different (capital) letters in each treatment indicate a significant difference between different days *p* < 0.05 according to Duncan test; Different lowercase letters in each day indicate that there is a significant difference between different treatments at *p* < 0.05 according to Duncan test; Values were expressed as means ± standard deviation (±SD).

**TABLE 16:** EΔ of the burger samples containing free and encapsulated essential oils of *E. platyloba* and basil.

Storage time (Day)				
Experimental treatments	1	4	8	12
C	0.0 ± 0.0	1.42 ± 0.01^C,a^	2.88 ± 0.10^B,a^	4.24 ± 0.09^A,a^
T1	0.0 ± 0.0	1.20 ± 0.10^C,ab^	1.82 ± 0.19^B,c^	3.21 ± 0.16^A,b^
T2	0.0 ± 0.0	1.19 ± 0.30^C,ab^	2.30 ± 0.28^B,b^	3.42 ± 0.07^A,b^
T3	0.0 ± 0.0	0.60 ± 0.07^C,de^	1.24 ± 0.04^B,d^	1.75 ± 0.14^A,d^
T4	0.0 ± 0.0	0.74 ± 0.13^C,cd^	1.50 ± 0.18^B,cd^	2.19 ± 0.15^A,c^
T5	0.0 ± 0.0	0.94 ± 0.07^C,bc^	1.78 ± 0.14^B,c^	2.48 ± 0.54^A,c^
T6	0.0 ± 0.0	0.41 ± 0.16^C,e^	0.92 ± 0.20^B,e^	1.35 ± 0.16^A,d^

*Note:* Significant different (capital) letters in each treatment indicate a significant difference between different days *p* < 0.05 according to Duncan test; Different lowercase letters in each day indicate that there is a significant difference between different treatments at *p* < 0.05 according to Duncan test; Values were expressed as means ± standard deviation (±SD).

Variations in brightness during storage may be due to structural changes in the meat particularly protein denaturation leading to greater light dispersion. The reduction in the raw meat redness is linked to oxidative reactions. Primary lipid oxidation products such as hydroperoxides and reactive oxygen species facilitate the oxidation of ferrous iron (Fe^2+^) in oxymyoglobin to ferric iron (Fe^3+^), resulting in metmyoglobin formation. Secondary lipid oxidation products like unsaturated aldehydes significantly contribute to metmyoglobin formation in fresh beef. Increased yellowing may indicate more brown coloration in the meat. The yellowing profile demonstrates the positive effect of the essential oils on browning. Jaundice progression can be linked to oxidative processes that create Schiff pigments like lipofuscins from lipid‐protein complexes (Elhadef et al. 2020). During meat storage, spoilage causes deoxymyoglobin (purple) and oxymyoglobin (red) to oxidize into metmyoglobin (brown). This biochemical change reduces the red color and increases yellowing, compromising the meat's visual appeal (Xiong et al. 2020). The increased redness, yellowness, and brightness in the essential oil treatments are likely due to higher phenolic compound concentrations. Certain phenolic compounds like anthocyanins are natural pigments that create color variations from red to blue based on pH levels. This mechanism may significantly redden the meat. The antioxidant properties of the bioactive substances in the essential oils may prevent oxidation in meat, potentially enhancing its red coloration (Elhadef et al. 2020; Xiong et al. 2020). Indeed, the bioactive substances in the essential oils significantly inhibit the oxidation of oxymyoglobin pigments in red meat. This inhibition causes the meat to change color from red to brown. Moreover, chitosan influences prolonged red coloration retention. Myoglobin, found in animal tissues and responsible for the reddish hue of raw meat, changes color to red when exposed to oxygen. The reduction in redness during storage is due to oxidative reactions of colored pigments from light exposure and the formation of gray‐brown metmyoglobin. Yellowing index variations during storage may relate to oxidation levels. Higher oxidation levels correlate with increased jaundice. The decrease in luminosity may be due to the degradation of similar pigments during storage.

Oxidation of meat reduces water retention in myofibrils, resulting in greater loss of meat juice. Lipid oxidation increases cell membrane permeability, reducing water retention in the product. This phenomenon can reduce brightness in the stored burger samples. The nano‐encapsulated treatments may preserve the product's water‐holding capacity and reduce oxidative processes (Khoshtinat et al. 2023). On the last day of refrigeration, the control sample had the highest Δ*E*. However, the nanocapsules containing the essential oil showed lower Δ*E* values. The application of the released essential oil affects the color retention on the burger's surface. This effect may be due to the antioxidant and antimicrobial activities of the essential oil's bioactive compounds.

A significant increase was noted in the control sample. Higher encapsulation efficiency of microcapsules correlates with lower Δ*E* values. Oxidation of myoglobin and oxyhemoglobin creates brown metmyoglobin, leading to reduced redness in meat. This biochemical process significantly affects color variation, impacting the Δ*E* value (Khoshtinat et al. 2023). Cerrón‐Mercado et al. (2022) reported that emulsions with the chincho (*Tagetes elliptica* Sm.) essential oil are beneficial in the burger samples. They also found that the brightness, redness, and yellowness increased while ΔE values decreased. Additionally, in another investigation, it was found that the *Allium jesdianum* extract increased redness, yellowness, and brightness in the meat samples. Storage duration negatively affected brightness and increased redness and yellowness (Ghorbani et al. 2024).

### 3.8 The Microbial Characteristics of the Burger Samples

The microbial characteristics, including total counts, *Staphylococcus aureus* , mold, yeast, and psychotropic bacteria are shown in Tables 17, 18, 19, 20. There were significant differences in microbial characteristics among the burger samples (*p* ≤ 0.05). The *E. coli* and *Salmonella* counts were zero in all treatment groups and storage intervals. The experimental treatments with *E. platyloba* and basil essential oils reduced total microbial counts, including *S. aureus* , molds, and yeasts in psychrotrophic bacteria. The *E. platyloba* essential oil treatments significantly reduced total microbial counts, *S. aureus* , molds, yeasts, and psychrotrophic bacteria compared to those using the basil essential oil. On day one of the study, *S. aureus* and yeast counts were zero in all treatment groups. As the storage period progressed, there was a noticeable increase in total counts of microorganisms, *S. aureus* , molds, yeasts, and psychrotrophic bacteria in all samples.

**TABLE 17:** The total microbial count (cfu/g) of the burger samples containing free and encapsulated essential oils.

Storage time (Day)				
Experimental treatments	1	4	8	12
C	4.347 ± 0.006^D,a^	6.124 ± 0.013^C,a^	9.402 ± 0.05^B,a^	11.385 ± 0.006^A,a^
T1	4.174 ± 0.017^D,e^	5.464 ± 0.012^C,c^	8.443 ± 0.17^B,c^	10.388 ± 0.006^A,c^
T2	4.270 ± 0.023^D,c^	5.645 ± 0.079^C,b^	9.058 ± 0.13^B,b^	10.967 ± 0.033^A,b^
T3	4.474 ± 0.015^D,c^	5.019 ± 0.017^C,f^	7.945 ± 0.13^B,e^	8.455 ± 0.012^A,f^
T4	4.306 ± 0.003^D,b^	5.271 ± 0.010^C,e^	8.140 ± 0.03^B,d^	9.452 ± 0.011^A,e^
T5	4.108 ± 0.022^D,f^	5.383 ± 0.004^C,d^	7.965 ± 0.06^B,e^	9.778 ± 0.049^A,d^
T6	4.224 ± 0.020^D,d^	4.709 ± 0.033^C,g^	7.377 ± 0.08^B,f^	7.911 ± 0.016^A,g^

*Note:* Significant different (capital) letters in each treatment indicate a significant difference between different days *p* < 0.05 according to Duncan test; Different lowercase letters in each day indicate that there is a significant difference between different treatments at *p* < 0.05 according to Duncan test; Values were expressed as means ± standard deviation (±SD).

**TABLE 18:** The average count of *Staphylococcus aureus* (cfu/g) of the burger samples containing free and encapsulated essential oils of *E. platyloba* and basil.

Storage time (Day)				
Experimental treatments	1	4	8	12
C	0.0 ± 0.0	4.406 ± 0.004^C,a^	5.382 ± 0.006^B,a^	7.366 ± 0.013^A,a^
T1	0.0 ± 0.0	3.769 ± 0.047^C,c^	4.472 ± 0.004^B,c^	6.348 ± 0.005^A,c^
T2	0.0 ± 0.0	4.227 ± 0.015^C,b^	4.959 ± 0.010^B,b^	6.639 ± 0.042^A,b^
T3	0.0 ± 0.0	3.218 ± 0.022^C,f^	4.123 ± 0.030^B,f^	5.872 ± 0.035^A,e^
T4	0.0 ± 0.0	3.401 ± 0.011^C,e^	4.350 ± 0.018^B,e^	6.290 ± 0.008^A,cd^
T5	0.0 ± 0.0	3.471 ± 0.003^C,d^	4.243 ± 0.021^B,d^	6.269 ± 0.008^A,d^
T6	0.0 ± 0.0	2.971 ± 0.016^C,g^	3.728 ± 0.073^B,g^	5.646 ± 0.068^A,f^

*Note:* Significant different (capital) letters in each treatment indicate a significant difference between different days *p* < 0.05 according to Duncan test; Different lowercase letters in each day indicate that there is a significant difference between different treatments at *p* < 0.05 according to Duncan test; Values were expressed as means ± standard deviation (±SD).

**TABLE 19:** The mean count of mold and yeast (cfu/g) of the burger samples containing free and encapsulated essential oils of *E. platyloba* and basil.

Storage time (Day)				
Experimental treatments	1	4	8	12
C	0.0 ± 0.0	3.754 ± 0.070^C,a^	5.250 ± 0.007^B,a^	7.357 ± 0.017^A,a^
T1	0.0 ± 0.0	3.032 ± 0.006^C,b^	4.535 ± 0.032^B,c^	6.913 ± 0.021^A,c^
T2	0.0 ± 0.0	3.104 ± 0.016^C,b^	4.952 ± 0.045^B,b^	7.169 ± 0.012^A,b^
T3	0.0 ± 0.0	2.816 ± 0.020^C,d^	4.023 ± 0.015^B,f^	6.257 ± 0.009^A,f^
T4	0.0 ± 0.0	2.932 ± 0.019^C,e^	4.295 ± 0.007^B,e^	6.361 ± 0.008^A,e^
T5	0.0 ± 0.0	2.924 ± 0.048^C,c^	4.411 ± 0.003^B,d^	6.460 ± 0.005^A,d^
T6	0.0 ± 0.0	2.515 ± 0.098^C,e^	3.889 ± 0.023^B,g^	5.869 ± 0.006^A,g^

*Note:* Significant different (capital) letters in each treatment indicate a significant difference between different days *p* < 0.05 according to Duncan test; Different lowercase letters in each day indicate that there is a significant difference between different treatments at *p* < 0.05 according to Duncan test; Values were expressed as means ± standard deviation (±SD).

**TABLE 20:** The average count of cychrotrophic bacteria (cfu/g) of the burger samples containing free and encapsulated essential oils of *E. platyloba* and basil.

Storage time (Day)				
Experimental treatments	1	4	8	12
C	3.455 ± 0.009^A,a^	5.098 ± 0.025^C,a^	8.350 ± 0.020^B,a^	9.539 ± 0.078^A,a^
T1	2.900 ± 0.059^A,e^	4.878 ± 0.017^C,a^	7.537 ± 0.092^B,c^	8.934 ± 0.052^A,c^
T2	3.243 ± 0.005^A,b^	5.048 ± 0.021^C,a^	7.963 ± 0.005^B,b^	9.318 ± 0.007^A,b^
T3	3.155 ± 0.014^A,c^	4.197 ± 0.024^C,b^	6.471 ± 0.007^B,e^	8.230 ± 0.012^A,f^
T4	3.281 ± 0.010^A,b^	4.398 ± 0.010^C,b^	7.276 ± 0.006^B,d^	8.334 ± 0.018^A,e^
T5	2.814 ± 0.064^A,f^	4.430 ± 0.004^C,b^	7.474 ± 0.008^B,c^	8.561 ± 0.066^A,d^
T6	3.036 ± 0.030^A,d^	3.655 ± 0.550^C,e^	6.129 ± 0.029^B,f^	7.650 ± 0.093^A,g^

*Note:* Significant different (capital) letters in each treatment indicate a significant difference between different days *p* < 0.05 according to Duncan test; Different lowercase letters in each day indicate that there is a significant difference between different treatments at *p* < 0.05 according to Duncan test; Values were expressed as means ± standard deviation (±SD).

The total microbial count is a key for evaluating a product's hygienic quality and its suitability for consumption. The lower microbial load in the essential oil treatments may be due to the bioactive substances like phenolic compounds (Bajalan et al. 2017; Ghasemi Pirbalouti, Setayesh, et al. 2013). These bioactive substances in the essential oils disrupt microbial membranes, degrading lipopolysaccharides. This disruption increases the permeability of the cytoplasmic membrane to ATP (Rezaei and Ghasemi Pirbalouti 2019). ATP depletion leads to cellular energy exhaustion and ultimately causes apoptosis or necrosis. The chemical structure of phenolic compounds significantly affects their antimicrobial mechanisms. Hydroxyl groups in phenolic compounds are crucial for the antimicrobial properties of the essential oils. An active hydroxyphenolic group promotes hydrogen bond formation with enzyme active sites. Flavonoids and flavonols may exert their effects through enzyme inhibition via interactions with sulfhydryl groups or nonspecific interactions with microbial proteins (Rezaei and Ghasemi Pirbalouti 2019). These compounds may form complexes with microbial cell walls or disrupt microbial cellular membranes (Mozafari et al. 2023).

Nanocapsules with plant essential oils reduced the microbial load more effectively than the control sample during storage. This observation highlights improved antimicrobial efficacy and a controlled release profile of the nanocapsules. It suggests that nanoencapsulation may enhance the antimicrobial properties of the essential oils (Mozafari et al. 2023). In nano‐encapsulated treatments, the low microbial load can be linked to the bioactive substances in the essential oils and the antimicrobial properties of chitosan. Chitosan's antimicrobial action mainly arises from the electrostatic interaction between its positively charged molecules and the negatively charged components of microbial cell membranes. This interaction alters membrane permeability and may affect complex formation on microbial cell surfaces. These changes hinder nutrient transport, limiting microbial growth. Chitosan effectively interacts with ionic groups on bacterial cell surfaces, forming polyelectrolyte complexes. This interaction hinders nutrient transport into cells, limiting their growth and proliferation (Foromandi and Khani 2023; Mahdavi et al. 2018; Zhang et al. 2020). Researchers found that the nanoencapsulated treatments had decreased the antibacterial activity during initial storage periods. This observation may be due to the gradual release of the essential oil from the nanoparticles.

The nanoencapsulation improves the surface area, stability, and dispersion of the essential oils. This technology disrupts microorganisms' biochemical and structural functions, impairing cellular viability and leading to cell death. Nanocapsules protect essential oils from evaporation and degradation, improve their solubility, and boost their antibacterial and antioxidant properties (Zhang et al. 2020). The rise in microbial population during refrigerated storage can be attributed to the proliferation of psychrophilic bacteria (Zhang et al. 2020). Investigators (Mozafari et al. 2023) claim that the nanoencapsulated treatments showed reduced antibacterial activity during initial storage. This may result from the gradual release of the essential oil from the nanoparticles.

Nano‐encapsulation enhances the surface area, stability, and dispersion of the essential oils. This technology disrupts microorganisms' functions, impairing viability and causing cell death. Nanocapsules boost the essential oils by preventing evaporation and degradation, improving solubility, and increasing the antibacterial and antioxidant properties (Mozafari et al. 2023). Meat products create ideal conditions for fungal growth. This is largely due to their high water activity. Sufficient nitrogenous compounds and a favorable pH range create adequate fermentable carbohydrate reserves. Molds and yeasts are aerobic microorganisms found on meat, showing greater resistance to antimicrobial agents like the essential oils than bacteria. The reduction in fungal growth in the samples with free essential oils and nanocapsules is due to the diverse secondary metabolites in the essential oils. These metabolites inhibit the growth of yeasts and molds, similar to their effects on bacteria (Mukurumbira et al. 2022; Zhang et al. 2021). Probably, the decrease in fungal populations in the treatments using both free and nanoencapsulated essential oils results from bioactive compounds like phenols, flavonoids, and phenylpropanoids in the studied essential oils (Moghaddam et al. 2015). These compounds can alter microbial cell wall proteins, denaturing specific enzymes. Additionally, they can form complexes with environmental nutrients like carbohydrates, proteins, vitamins, and minerals, making them inaccessible to microorganisms. For instance, the phenolic compounds exhibit antimicrobial properties by disrupting lipid membrane permeability in cell walls and mitochondria. This disruption can lead to cell death by expelling cellular contents or inhibit growth and proliferation.

The hydrophobic characteristics of the active constituents facilitate the release of phenolic compounds into the cells. The concentration of the phenolic compounds decreases during storage, leading to reduced antimicrobial activity. This reduction in antimicrobial efficacy leads to increased fungal growth, diminishing the essential oil's effectiveness in later storage stages (Mozafari et al. 2023).

Nanocapsules containing the essential oils have showed improved effectiveness in reducing microbial load. This trend indicates a notable boost in antimicrobial effectiveness from the controlled release of nanocapsules and improved properties of the essential oil after nanoencapsulation (Mozafari et al. 2023). Furthermore, in the context of nano‐encapsulated treatments, the observed low microbial load is attributed to the independent action of phenolic compounds present in the essential oil, which is distinctly influenced by the antimicrobial properties of chitosan. The inhibitory mechanism of chitosan molecules on microbial growth is primarily attributed to the interaction between the positively charged regions of chitosan and the negatively charged components of microbial cell membranes. This interaction results in alterations to membrane permeability and affects the capacity of microbial cells to form surface complexes, thereby hindering the transport of essential nutrients into the cells. Chitosan possesses the capability to interact with ionic groups present on the surface of bacterial cells, thereby facilitating the formation of polyelectrolyte complexes with surface compounds on these cells. This interaction effectively hinders the transfer of nutrients into the bacterial cells, ultimately inhibiting their growth (Foromandi and Khani 2023, Mahdavi et al. 2018, Zhang et al. 2020). Reduction sizes in chitosan nanocapsules incorporating basil and *E. platyloba* essential oils may have a particular facility to pass through microbial cells and interfere with vital cell processes (Lelis et al. 2021). The wall material in the nanocapsulation can interact with the surface of the bacterial cell such as inter‐membrane transfer, release contact, absorption, fusion, and phagocytosis and form pores, which facilitates the action of the antibacterial and at the same time increases the permeability of the cell, in addition to allowing continuous diffusion of antibacterial constituents across the cell membrane (Lelis et al. 2021). Additionally, chitosan nanocapsules can easily permeate porous proteins in the bacterium's outer membrane, facilitating essential oils delivery. Results of studies illustrated that increases in essential oils loading with chitosan can damage the bacterial cell morphology and exhibit irregular, deformed, and incomplete structures.

Researchers have reported that the *E. platyloba* essential oil presents a potential alternative antifungal agent for the replacement of synthetic chemical preservatives in food products (Ghasemi Pirbalouti, Setayesh, et al. 2013; Moghaddam et al. 2015). The main contaminants in the meat products are Enterobacteriaceae, particularly *E. coli* and *Salmonella*. These bacteria produce biogenic amines as metabolic byproducts. These compounds are linked to foodborne illnesses and reduce product quality. A key health indicator for evaluating meat safety is the measurement of *E. coli* bacteria. The presence of *E. coli* and *Salmonella* makes meat products unsafe to eat. Gram‐negative bacteria are often seen as resistant to natural antimicrobials due to their outer membrane characteristics. Incorporating nanoencapsulated essential oils has proven effective in reducing this bacterial group (Mozafari et al. 2023). Results of a previous investigation indicated that the microencapsulated basil essential oil significantly reduced *E. coli* and *Salmonella* in the sauce samples (Ozdemir et al. 2021). In current research, the essential oil treatments significantly reduced the microbial counts compared to the control group. Additionally, the nano‐encapsulated treatments showed a lower microbial load during storage compared to free essential oil treatments and the control group. The study found no presence of *Salmonella* bacteria in any samples on any observation day. Similar findings have been recorded for cryophilic bacteria (Mozafari et al. 2023).

### 3.9 Chemometric Analysis

**FIGURE 5:**
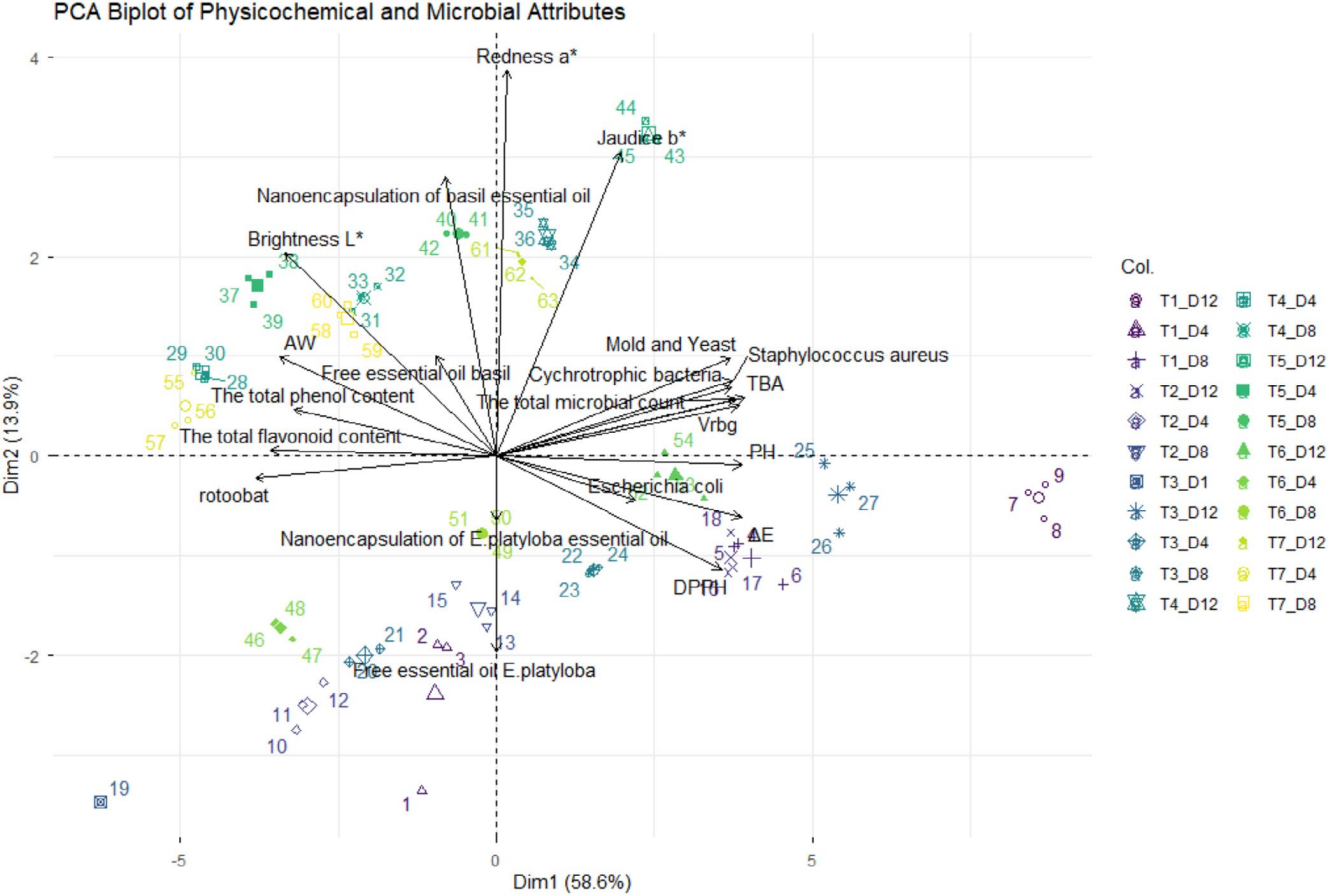
Principal component analysis (PCA) of the burger samples containing free and encapsulated essential oils.

Principal component analysis (PCA) was performed to evaluate the overall pattern of changes in the physicochemical, antioxidant, microbial, and qualitative characteristics of the burger samples during storage and under the influence of different treatments of free and nanoencapsulated essential oils of two herbs. The main two principal components explained a significant part of the data variation, indicating the high power of this model in distinguishing treatments. In the Biplot display, the samples were clustered based on the type of treatment (free, nanoencapsulated with different concentrations or ratios of the essential oils) and the day of storage (Figure 5). Fresh samples (day‐1) were generally located in the area defined by vectors related to positive quality attributes including moisture, color, textural properties, antioxidant capacity (DPPH), total phenols, and total flavonoids, as well as low microbial load. This indicates that at the beginning of storage, the differences among the treatments were mainly due to the initial quality and structure of the formulation.

Indeed, the samples treated with chitosan nanoencapsulation generally located in the region that correlated with higher values of antioxidant parameters and favorable sensory and textural properties. This situation shows the effective role of nanoencapsulation in the stability of the essential oil active compounds and their controlled release during storage. On the other hand, the samples treated with free essential oil (without encapsulation) approached areas associated with increased TBA, color changes (high ΔE), moisture loss, and increased microbial load over time, indicating a decrease in the efficiency of antioxidant protection over time. The samples from the final days of storage (Day 12, 8 or 4 depending on the design) were clearly oriented towards vectors related to microbial counts (TPC, molds and yeasts, *S. aureus* , *E. coli* ) and spoilage indicators (such as TBA and color changes). This pattern confirms that PCA was able to clearly distinguish the different stages of spoilage and the nanoencapsulated treatments were able to keep the samples in their associated spaces with higher quality and greater stability (Popa et al. 2021).

In general, PCA analysis showed that (1) the type of treatment had a decisive effect on the stability and quality of the product; (2) the nanoencapsulated treatments, particularly the samples containing nanoencapsulated essential oils with chitosan, had the greatest distance from the control group and free essential oil, and also they had the highest overlap with positive quality vectors; (3) increasing the number of days of storage had the dominant influence in transferring the samples from the desired quality zone to the spoilage zone; however, this trend was significantly slowed down in the nanoencapsulated treatments.

**FIGURE 6:**
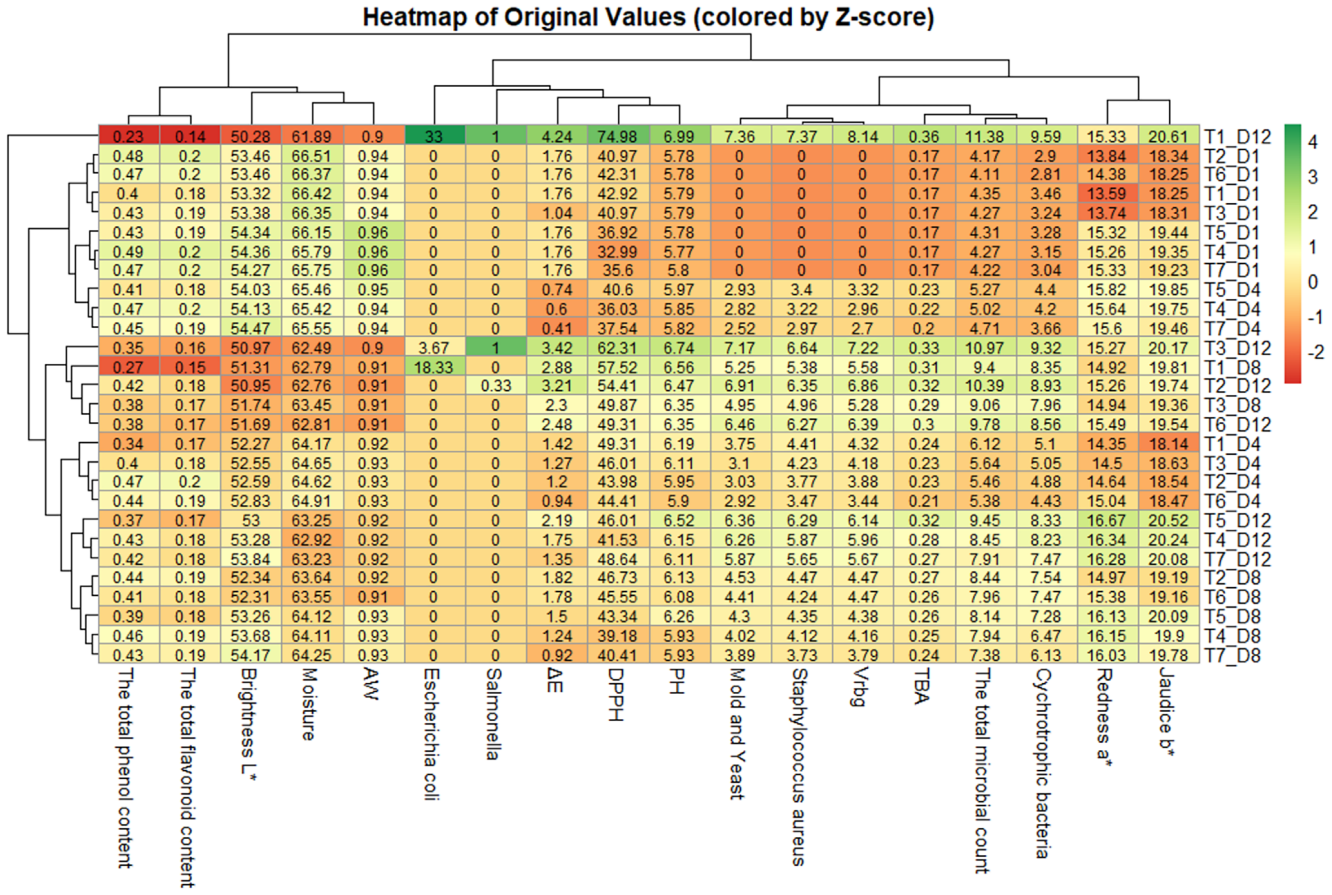
Hierarchical clustering analysis (HCA) heatmap of the burger samples containing free and encapsulated essential oils.

Hierarchical cluster analysis (HCA) with a Heat map graph was drawn to display the simultaneous pattern of changes in various characteristics of the samples in a single screen and to discover similarities and differences among treatments and storage periods (Figure 6). The clustering pattern showed that the samples were divided into two main clusters:

- The first cluster consisted of high‐quality treatments with favorable physicochemical, antioxidant, and antimicrobial properties, mainly including the samples in early days of storage, and especially the samples treated with chitosan nanoencapsulation. This group had characteristics such as low TBA levels, high color stability, strong antioxidant activity, and low microbial load. Warm colors in this region indicate the presence of high amounts of phenolic compounds, flavonoids, and antioxidant factors.
- The second cluster mainly consisted of control groups, free essential oil, and end‐of‐storage samples, which were characterized by features such as increased microbial load (TPC, mold and yeast, *Staphylococcus*), increased TBA, decreased antioxidant activity, noticeable color changes, and decreased textural quality. The presence of bluish‐green colors in this area indicates a severe decrease in quality and increased oxidative and spoilage processes.

Overall, the Heat map graph indicated that (1) treatment (type of essential oil and encapsulation method) was the most important determinant of clusters; (2) chitosan nanoencapsulated treatments performed best in terms of overall quality; (3) the spoilage trend in the control and free essential oil groups was clearly visible, and clustering showed this pattern with very high accuracy; (4) the positive correlation between high antioxidant activity and reduced microbial spoilage indices in the nanoencapsulated samples was clearly evident.

Finally, according to results of HCA demonstrated that the nanoencapsulated samples clustered close to positive characteristics throughout all days of storage, however, the free essential oil and control treatments clearly tended towards spoilage and quality degradation characteristics. These differences indicate the key role of nanoencapsulation in boosting antioxidant stability, limiting microbial growth, and maintaining the physicochemical properties of burgers during storage.

## 4 Conclusions

Nanoencapsulation with the essential oils of basil and *E. platyloba* produced nanocapsules with small particle sizes, high encapsulation efficiency, and stable zeta potentials. The burger samples that incorporated the *E. platyloba* and basil essential oils demonstrated a notable reduction in TBA, overall microbial counts, and specific pathogens including *Staphylococcus aureus* , molds, yeasts, and psychrotrophic bacteria. In conclusion, the nanoencapsulation with the essential oils of basil and *E. platyloba* is recommended to increase the shelf life of burgers. Further studies are needed to evaluate the sensory evaluation of the burgers, isolation and purification of secondary metabolites of the essential oils, as well as toxic impacts of nanoencapsulated bioactive compound formulations and to monitor the implementation of regulations on nanoparticles currently in use.

## Author Contributions

**Samira Malekiazar:** investigation, writing – original draft, resources. **Alireza Rahman:** conceptualization, writing – review and editing, project administration, supervision. **Mohammadreza Khani:** conceptualization, visualization, project administration, supervision, data curation. **Toktam Mostaghim:** validation, methodology, formal analysis. **Abdolah Ghasemi Pirbalouti:** writing – review and editing, methodology, software, data curation.

## Conflicts of Interest

The authors declare no conflicts of interest.

## Data Availability

The data that support the findings of this study are available from the corresponding author upon reasonable request.
